# Advancements in binary and ternary transition metal-based composites for high-performance supercapacitors: a comprehensive review

**DOI:** 10.1039/d5ra00528k

**Published:** 2025-03-25

**Authors:** Jannatun Zia, M. S. S. R. Tejaswini

**Affiliations:** a Department of Chemistry, School of Engineering, Siddhartha Academy of Higher Education Deemed to be University Vijayawada A. P. India jannat22oct@gmail.com; b Department of Chemistry, School of Applied Sciences and Humanities, Vignan's Foundation for Science, Technology and Research Vadlamudi Guntur A. P. India

## Abstract

As the demand for efficient and high-performance energy storage devices continues to rise, supercapacitors have emerged as a promising technology due to their rapid charge–discharge capabilities and long cycle life. Among the various strategies to enhance supercapacitor performance, binary and ternary transition metal-based composites have garnered significant attention. These composites offer a unique approach by combining multiple transition metals, which synergistically enhance electrochemical performance through both physical and chemical charge storage mechanisms. This review provides an in-depth analysis of the latest research on binary and ternary transition metal composites, discussing their electrochemical properties, synthesis methods, and performance metrics in supercapacitor applications. The combination of different transition metals in composite materials as energy storage electrodes allows for a broader voltage window, increased energy density, enhanced power density, and improved cycling stability. Additionally, we discuss the structural and morphological features of these composite materials, such as porosity, surface area, and conductivity, which play critical roles in determining overall performance. Furthermore, the review highlights the challenges faced in optimizing these composites, including material scalability, cost-effectiveness, and long-term stability. The paper also outlines future research directions, emphasizing the potential of binary and ternary transition metal-based composites in supercapacitor applications, providing insights into potential avenues for the next generation of high-performance energy storage systems. This review thus provides valuable insights into both the current state and future potential of these composite materials in high-performance supercapacitors.

## Abstract 2

As the demand for efficient and high-performance energy storage devices continues to rise, supercapacitors have emerged as a promising technology due to their rapid charge–discharge capabilities and long cycle life.

## Graphical abstract

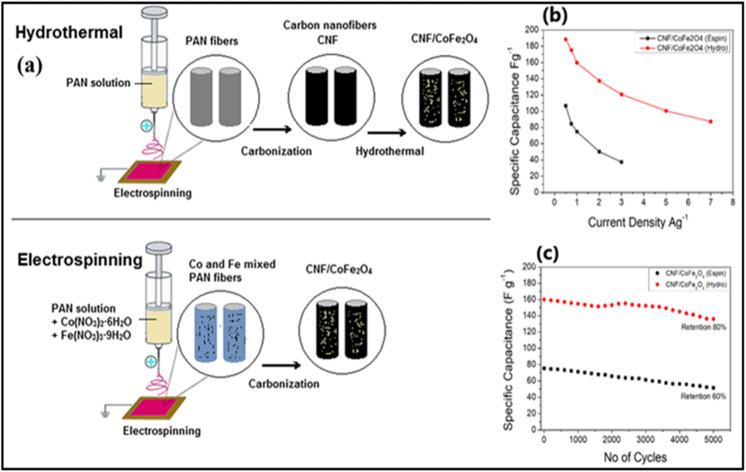

## 1. Introduction

The global energy consumption from fossil fuels reached 13.731 billion tons of oil equivalents (BTOE) in 2012, with projections rising to 18.30 BTOE by 2035. This growth, coupled with diminishing fossil fuel reserves, has led to economic challenges, such as volatile prices and supply chain disruptions. Moreover, the widespread burning of fossil fuels has significantly increased carbon dioxide emissions, contributing to global warming and environmental changes. Consequently, there is an urgent need for sustainable energy solutions, such as renewable energy sources and energy storage technologies.^1,2^

Lithium-ion batteries (LIBs), widely used in portable electronics and electric vehicles, offer high energy density but suffer from limited cycle life, suboptimal performance, and safety risks. Recent literature studies have reported that supercapacitors (SCs) can be a potential substitute for existing LIBs. Supercapacitors are among the most promising energy storage devices due to their exceptional power density, long cycle life, and fast charge–discharge capabilities. With growing global demands for efficient energy storage systems, supercapacitors have gained significant attention for applications in portable electronics, electric vehicles, renewable energy storage, and more. These devices offer significantly higher power density (*P*_d_), about 10 times that of LIBs, and can sustain tens of thousands to millions of charge and discharge cycles with minimal degradation, making them a safer option.^2,3^ The rapid development of SCs technology is further evidenced by their application in Airbus A380 aircraft.^4^ SCs are categorized based on their charge storage mechanisms and tailored for specific applications. Academic researchers and industries are focusing on various types of SCs to fulfil different energy storage requirements, each offering distinct advantages and uses.^5^ However, to further enhance their energy storage performance, there is a continuous need for innovative materials that can provide higher energy density, stability, and efficiency.

As the demand for enhanced SCs grows, researchers are investigating advanced electrochemical and nanotechnology approaches. The electrode material is a critical determinant, as it significantly affects the device's energy storage performance. Accordingly, it necessitates the utilization and exploration of novel materials with superior electrochemical properties. By developing innovative materials, sophisticated electrode designs, and cutting-edge manufacturing methods, researchers are pushing the boundaries of SCs technology, aiming for higher energy densities, faster charging times, and overall enhanced performance.^1,6^

To overcome this challenge, researchers have focused on improving the performance of supercapacitors through the development of advanced electrode materials. Transition metal-based composites (TMCs) have emerged as promising candidates due to their excellent electrochemical properties, including high electrical conductivity, large surface area, and favourable charge storage mechanisms. These composites, which often combine transition metals with other materials such as metal oxides, hydroxides, chalcogenides, phosphides, phosphates, carbon, and conducting polymers, can provide significant improvements in the energy storage capabilities of supercapacitors.^7^

There are two main types of transition metal-based composites: binary and ternary. Binary transition metal-based composites consist of two different electrode materials and have demonstrated significant progress in enhancing electrochemical performance by optimizing the synergistic effects between different metal ions. These binary systems can achieve better conductivity, electrochemical stability, and higher specific capacitance, which are critical for improving overall energy storage performance. On the other hand, ternary composites incorporate three different electrode materials into a single electrode and have the potential to offer even greater improvements in supercapacitor performance. By combining transition metals with carbon materials or conducting polymers, ternary composites can enhance conductivity, structural stability, and charge storage capacity, making them ideal for high-performance supercapacitor applications. This strategic combination enhances the efficacy and potency of energy storage systems, making composite electrodes a key focus in current research and development.^8^

**Scheme 1:**
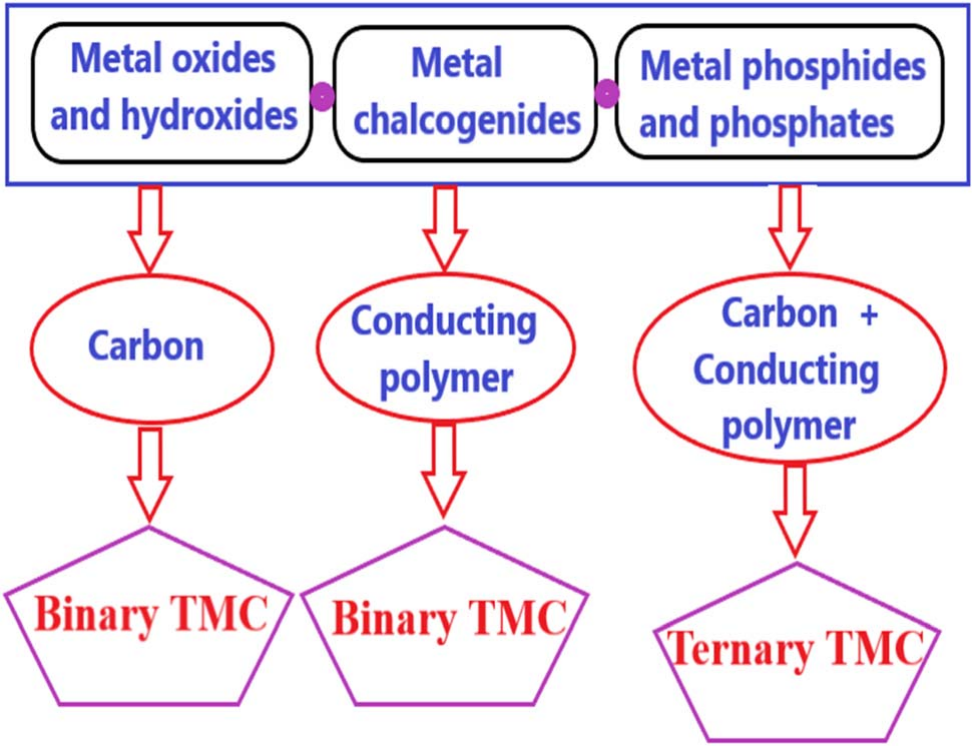
Schematic representation of possible combinations for the fabrication of transition metal composites for electrodes of SCs.

This review highlights recent advancements in binary and ternary transition metal-based composites (TMCs), including metal oxides, hydroxides, chalcogenides, phosphides, and phosphates, combined with carbon or conducting polymers to form binary composites. It examines their design, synthesis, structural properties, and electrochemical performance in supercapacitor applications. The review also explores ternary composites that incorporate both metals and conducting polymers with carbon (Scheme 1). A key focus is on the electrochemical performance of these composites, with a comprehensive analysis of various material combinations and their impact on energy storage efficiency. We aim to provide a comprehensive understanding of their potential for developing next-generation high-performance supercapacitors.

## 2. Binary transition metal composites for SCs

Binary transition metal composites, comprised of two distinct materials, encompass combinations such as metal oxides, hydroxides, chalcogenides, phosphides, and phosphates in conjunction with carbon or conducting polymers.

In systems for energy conversion and storage, carbon materials are reported to be extremely valuable. Their superior electrical conductivity, high surface-to-volume ratio, and robust cycling stability make them efficient for effective charge and discharge procedures. Their versatility is evident in their various allotropes such as graphene, fullerene, and graphite, and their wide range of structural forms, from powders and sheets to aerogels, fibers, and composites.^9,10^ Additionally, they are chemically stable, easy to process, non-toxic, lightweight, and possess adjustable porosity for enhanced performance. These materials encounter issues like low energy density (*E*_d_) and specific capacitance (*C*_s_). On the other hand, the high-power densities of electrodes made from transition metal compounds are often limited by their inherently poor conductivity and slow redox reaction kinetics. Integrating carbon materials with transition metal compounds has demonstrated enhanced electrochemical properties.^11^ This improvement is attributed to synergistic effects that create efficient pathways for electron and ion transfer, while also maintaining the structural stability of the entire electrode. The advantages are as follows:^11–13^

(1) The incorporation of carbon materials within transition metal compounds-based systems capitalizes on their exceptional conductivity, thereby circumventing the intrinsic constraints of transition metal compounds and promoting faster charge transport, culminating in enhanced electrochemical performance.

(2) Carbon functions as a structural framework, mitigating the agglomeration of transition metal compounds. This is pivotal, as agglomeration can result in an uneven distribution and a diminished active surface area, adversely affecting the electrochemical performance.

(3) The integration of transition metal compounds within a carbon matrix bolsters their thermal and chemical stability. Carbon materials, inherently inert and resistant to degradation, safeguard the transition metal compounds from environmental influences that could compromise their long-term performance.

(4) The inherent flexibility and lightweight characteristics of carbon accommodate the expansion and contraction that occur during charge and discharge cycles without compromising structural integrity. This adaptability reduces mechanical stress on the transition metal compounds, leading to longer life cycles and improved efficiency.

(5) Carbon materials' immense and broader surface area makes it easier for charge storage to occur through the creation of double layers, leading to elevated *P*_d_ and superior rate performance, in conjunction with the intrinsic pseudocapacitive contribution from transition metal compounds.

### 2.1 Carbon-based binary TMC

Transition metal oxides and hydroxides show significant potential for high-level energy storage, particularly as SCs electrode materials. As pseudo-capacitance and battery-type electrode materials, transition metal oxides offer higher *E*_d_ compared to carbon materials. Their redox properties ensure greater *E*_d_ and enhance electrochemical stability. These unique characteristics arise from their ability to exhibit various oxidation states, which facilitate rapid faradaic reactions. Moreover, they are capable of transiting between several oxidation states during charging and discharging owing to this functionality, thereby enabling quick movement of electrolyte ions into and out of the oxide lattice. Additionally, they are cost-effective, environmentally friendly, and possess high theoretical capacitance.^14–16^ Transition metal hydroxides are also extensively studied as SCs electrode materials due to their layered structure and high theoretical *C*_s_, with common examples being cobalt hydroxides, nickel hydroxides, and layered double hydroxides. However, their electrical conductivity and stability need further improvement, which can be accomplished by constructing various composite electrode material structures.^14^

**Fig. 1:**
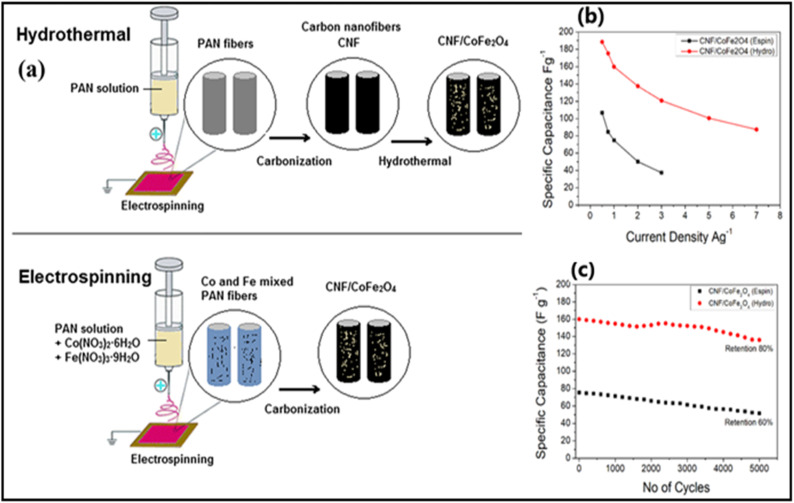
(a) Schematic representation of CNF/CoFe_2_O_4_ utilizing hydrothermal and electrospinning process, (b) variation of *C*_s_ with *I*_d_ and (c) cycling stability of CNF/CoFe_2_O_4_ utilizing hydrothermal and electrospinning process (reproduce from ref. 17 with permission from Springer© 2021).

In our interesting work on activated carbon nanofibers/cobalt ferrite (CNF/CoFe_2_O_4_) composites fabricated using electrospinning and hydrothermal methods (Fig. 1), the binary transition metal oxide-based carbon nanofibers composites are reported.^17^ It is concluded there that the hydrothermally prepared CNF/CoFe_2_O_4_ composite had a more extensive area in the cyclic voltammetry (CV) curve compared to the electro spun composite, indicating better electrochemical performance. Specifically, the hydrothermally synthesized composite successfully attained a *C*_s_ of 188.36 F g^−1^ computed from galvanostatic charge–discharge (GCD), whereas the electro spun composite had a lower *C*_s_ of 106.59 F g^−1^ at a current density (*I*_d_) of 0.5 A g^−1^. Additionally, the hydrothermal method resulted in 80% *C*_s_ retention, which was higher than the 60% retention observed with the electrospinning method. This superior performance stems from the higher carbon concentration in the hydrothermally synthesized composite and the fact that the CoFe_2_O_4_ nanoparticles were anchored to the CNF surface, rather than embedded within it as in the electro spun composite. This surface decoration in the hydrothermal method allowed for greater surface area exposure of both CoFe_2_O_4_ and CNF, facilitating better electrolyte penetration and overall superior electrochemical behavior. Consequently, the CNF/CoFe_2_O_4_ composite prepared *via* hydrothermal synthesis demonstrated more favourable characteristics which could be efficiently utilized as electrodes in SCs.

The past literature studies Zhao *et al.*^18^ reported the synthesis of ultra-fine RuO_2_ quantum dots on a reduced graphene oxide (rGO) surface with the help of microwave-assisted hydrothermal method. They investigated the impact of different RuO_2_ loadings on the electrode performance of the RuO_2_/rGO nanocomposite. The composite RG-2 (38 wt% RuO_2_ loading) demonstrated a *C*_s_ of 1120 F g^−1^ at a *I*_d_ of 1 A g^−1^. Additionally, it exhibited an excellent capacity retention rate of 84% as the *I*_d_ boosted from 1 A g^−1^ to 10 A g^−1^, along with outstanding cycling stability, retaining 89% of its capacity after 10 000 cycles. Chong Sun *et al.*^19^ synthesized a Ni–Co bimetallic hydroxide/multi-walled carbon nanotube (La–NiCo LDH/MWCNT) composite doped with varying concentrations of La^3+^ ions using a hydrothermal technique. At *I*_d_ of 1 A g^−1^, the sample with a 5% La^3+^ ion concentration exhibited a maximum *C*_s_ of 4396 F g^−1^. The *C*_s_ retention of this sample was 70.31% after 3000 cycles.

Metal chalcogenides, represented as M_*x*_C_*y*_ where M stands for transition metals from group 3 to 12 (including Ni, Co, Fe, Cu, Zn, Sc, Ti, V, Cr, W, Mo, *etc.*) and C represents chalcogens like sulfur (S), selenium (Se), and tellurium (Te), have been the subject of much scholarly inquiry. This interest is due to their versatile electronic structures, robust structural stability, anisotropic properties, adjustable kinetics, and remarkable electrochemical characteristics.^20^ These materials, which are composed of transition metal cations (M) and chalcogen anions, have structural variety that makes it possible to create adaptive surface and interfacial functions that improve ion intercalation in semiconductor conversion as well as storage of energy devices.^21^ These compounds consist of transition metal cations (M) and chalcogen anions (C). While interlayer bonding is governed by van der Waals forces, the bonds between metals and chalcogens are covalent. The metal atoms typically engage in bonding states with four electrons, resulting in an oxidation state of +4 for the metal and −2 for the chalcogen. The arrangement of C–M–C layers and the metal coordination can vary depending on the specific metals and chalcogens involved, leading to different polymorphic structures. Notably, the most common polymorphs are designated as 1T, 2H, and 3R, where the number denotes the layers within the crystalline unit cell and the letter indicates the symmetry of the structure—tetragonal (T), hexagonal (H), or rhombohedral (R).^21,22^ Due to their low toxicity and abundance, metal chalcogenides are economically viable materials for use as electrodes in SCs. They also find a wide range of applications in consumer electronics, backup power systems, hybrid electric vehicles, and more. However, the inherent properties of single-component metal chalcogenides, such as chemical and thermal stability and electrical conductivity, may not always meet the stringent requirements of emerging energy systems. To address these challenges, combining metal chalcogenides with other materials has been found to enhance their properties. The development of composite metal chalcogenides has demonstrated synergistic capabilities, maximizing their potential efficiency and performance.^23,24^

**Fig. 2:**
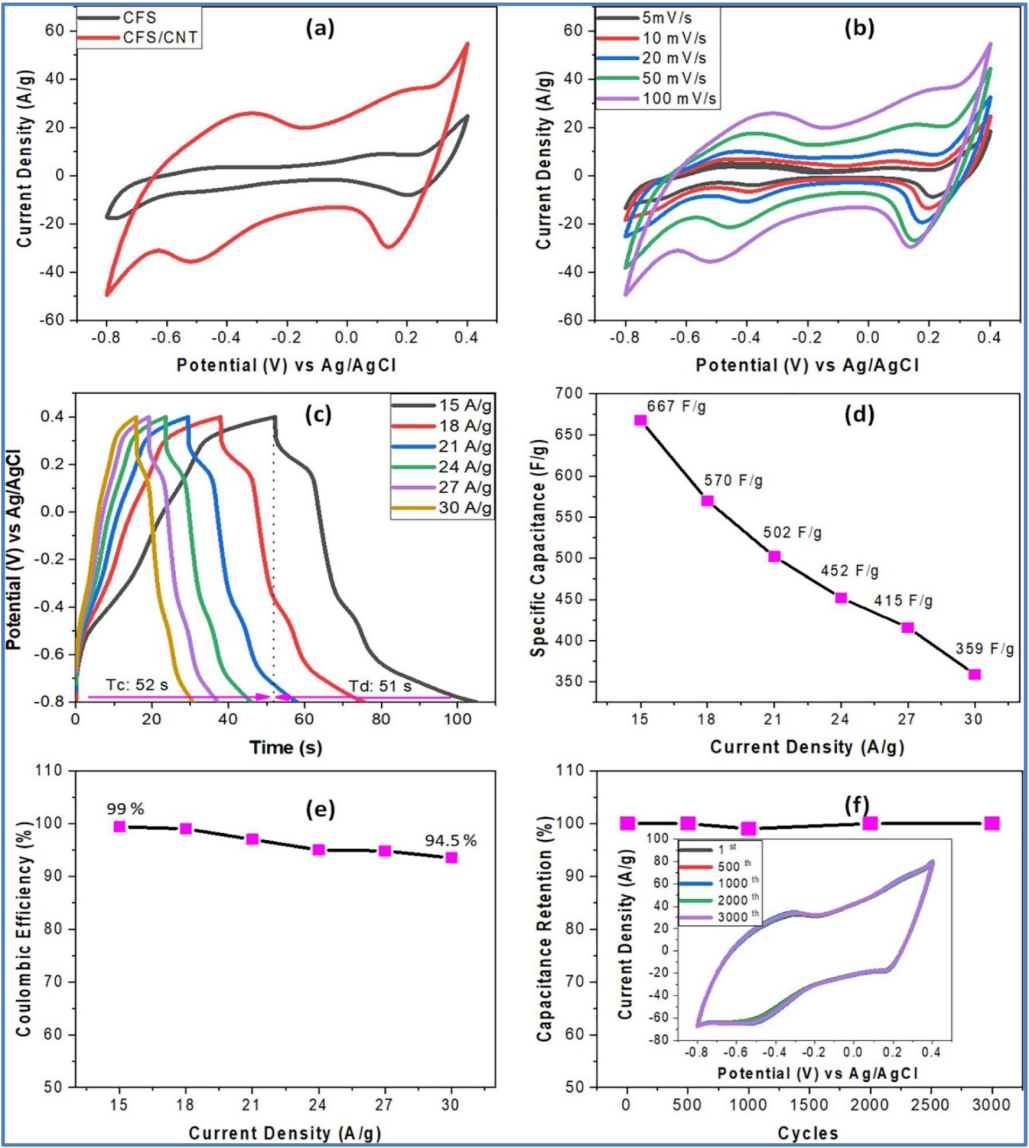
(a) The CV of the CFS and CFS/CNT composite electrode at 100 mV s^−1^ scan rate (*ν*), (b and c) the CV and the corresponding GCD plot of the CFS/CNT composite electrode at varied *ν* and varied *I*_d_, respectively, (d and e) the *C*_s_ and the *η* plot to the varied *I*_d_, respectively and (f) the cyclic stability plot for 3000 cycles (inset shows the CV curves at different cycles) of the CFS/CNT composite electrode (reproduced from ref. 25 with permission from Romania inoe© 2023).

Lokhande *et al.*^25^ synthesized a sulfide-based carbon composite electrode material, specifically CuFeS_2_/carbon nanotubes (CFS/CNT), through a hydrothermal method. The electrochemical studies as shown in Fig. 2 demonstrated that the composite electrode exhibited pseudocapacitive behavior, with enhanced electrical and charge transport properties. The CFS/CNT composite electrode achieved a high *C*_s_ of 667 F g^−1^ at a *I*_d_ of 15 A g^−1^ in a 1 M Na_2_SO_4_ electrolyte. Additionally, the electrode showed a high coulombic efficiency (*η*) of 95% and maintained 100% cyclic stability over 3000 cycles. To assess its practical application potential, a solid-state symmetric device (CFS/CNT//CFS/CNT) was created using a polymer gel electrolyte (PVA-Na_2_SO_4_). This device achieved a maximum *C*_s_ of 128 F g^−1^, an *E*_d_ of 22 W h kg^−1^, a *P*_d_ of 2083 W kg^−1^, and exhibited excellent durability with 94% cyclic stability over 10 000 cycles, making it a strong candidate for future energy storage.

Pandit *et al.*^26^ synthesized hexagonal VS_2_ nanoparticles on a multi-walled carbon nanotube (MWCNT) matrix using a combination of dip-and-dry and successive ionic layer adsorption and reaction (SILAR) methods. The resulting VS_2_/MWCNTs electrode demonstrated a high *C*_s_ of 830 F g^−1^ at a *ν* of 2 mV s^−1^, along with excellent stability, retaining 95.9% of its initial *C*_s_ over 10 000 cycles. The MWCNTs, processed by the dip-and-dry method, provided a structural and conductive framework, serving as the core for the VS_2_ nanostructure shell. This modified conductive network facilitated efficient faradaic charge transfer, while the prevention of charge accumulation helped maintain the structural integrity of the VS_2_ during charge–discharge (CD) cycles. Consequently, the system exhibited swift ion migration and improved CD kinetics at the conductive interface between VS_2_ and MWCNTs, reducing overall resistance. A flexible solid-state SSC device was fabricated using VS_2_/MWCNTs electrodes and a low-cost PVA-LiClO_4_ gel electrolyte. The SSC achieved a peak *C*_s_ of 182 F g^−1^ at a *ν* of 2 mV s^−1^, with a specific energy of 42 W h kg^−1^ and remarkable stability, retaining 93.2% of its *C*_s_ over 5000 cycles. As a demonstration of its practical application, the SSC successfully powered a panel labeled ‘VNIT’, consisting of 21 red LEDs.

Kirubasankar *et al.*^27^ developed a nanocomposite made of nickel selenide (NiSe) nanoparticles on graphene nanosheets (G) using an *in situ* hydrothermal approach. The NiSe nanoparticles were uniformly distributed throughout the graphene, creating a nanohybrid with improved diffusion and charge transport capabilities, in addition to many electrochemical active sites. As a NiSe–G nanohybrid combined with freestanding electrode, it showed outstanding electrochemical performance with a 98% retention of *C*_s_ after 2500 cycles and a high *C*_s_ of 1280 F g^−1^ at *I*_d_ of 1 A g^−1^. Additionally, an ASC device was put together using an electrospun PVdF membrane bathed in 6 M KOH as the electrolyte and separator, the NiSe–G nanohybrid as the PE, and AC as the NE. High *E*_d_ of 50.1 W h kg^−1^ and *P*_d_ of 816 W kg^−1^ were attained by this device at an extended operating voltage of 1.6 V.

**Fig. 3:**
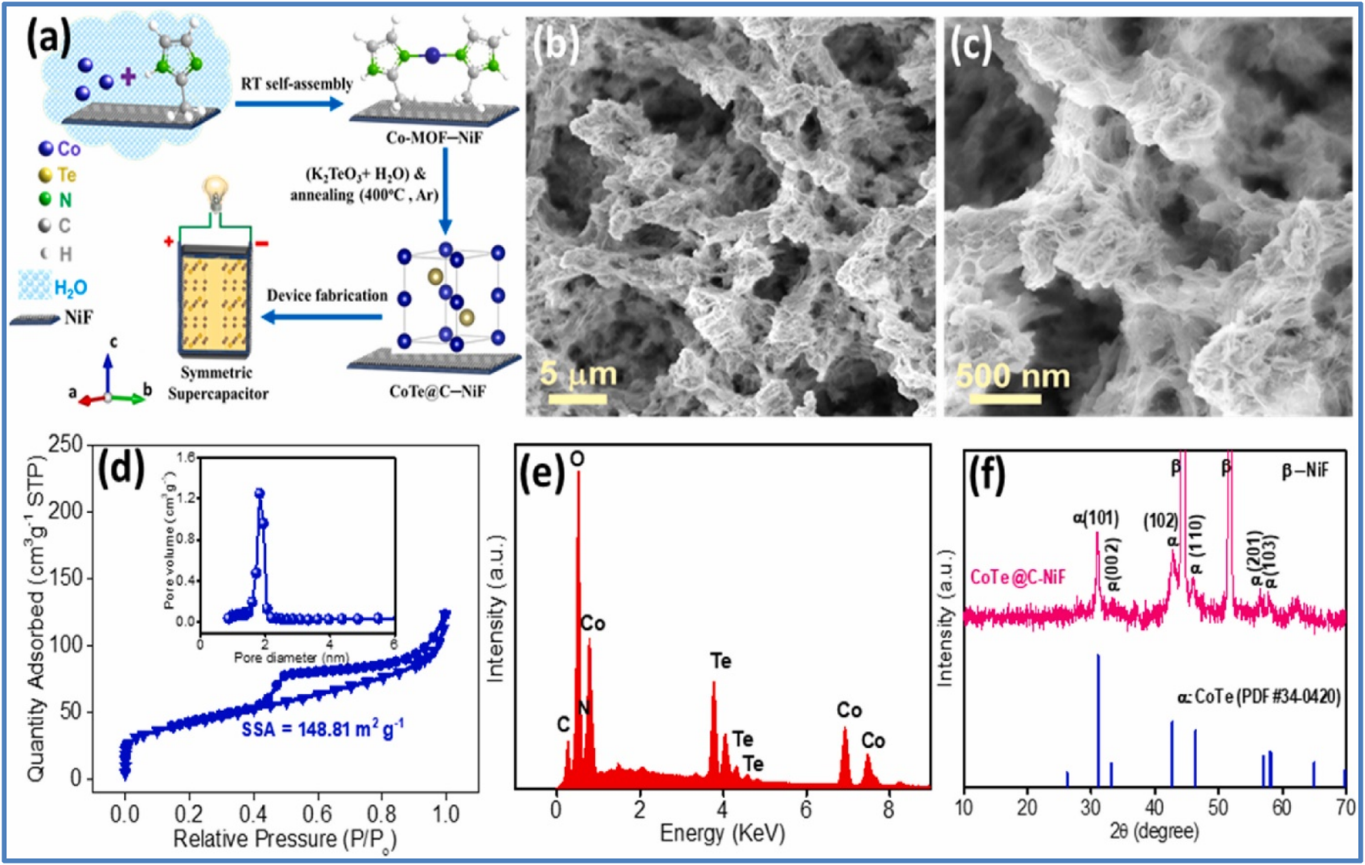
(a) Schematic representation of the synthesis of CoTe@C–NiF hybrid electrode; (b and c) SEM images; (d) N_2_ adsorption–desorption isotherms with pore size distribution curve in the inset; (e) EDS; and (f) XRD of CoTe@C–NiF hybrid electrode (reproduced from ref. 28 with permission from Elsevier© 2021).

**Fig. 4:**
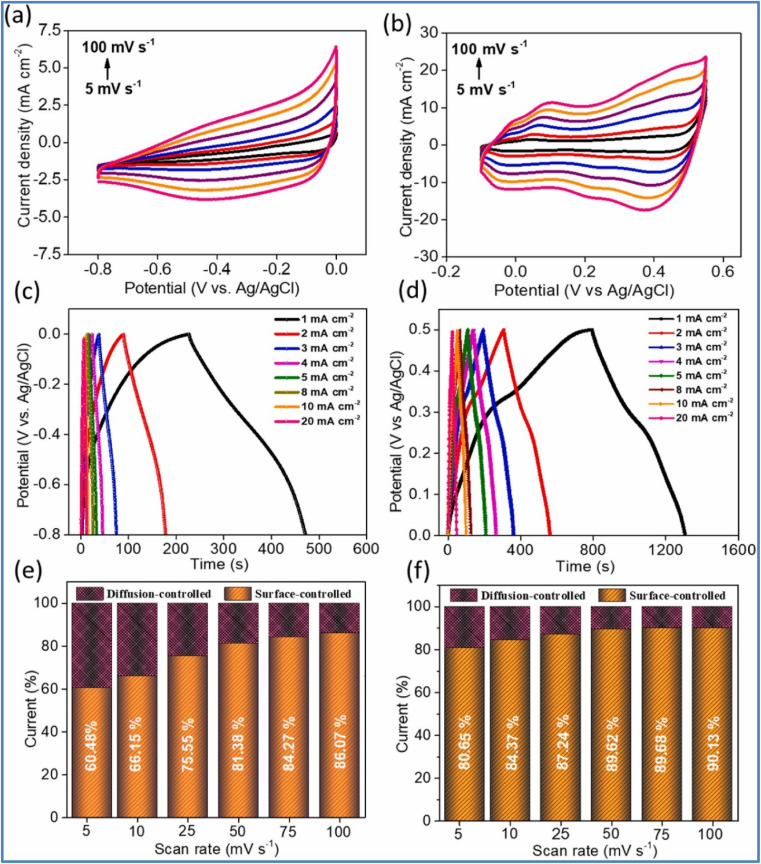
(a and b) CV curve; (c and d) GCD; and (e and f) current contribution curve of CoTe@C–NiF in the negative and positive potential window, respectively (reproduced from ref. 28 with permission from Elsevier© 2021).

**Fig. 5:**
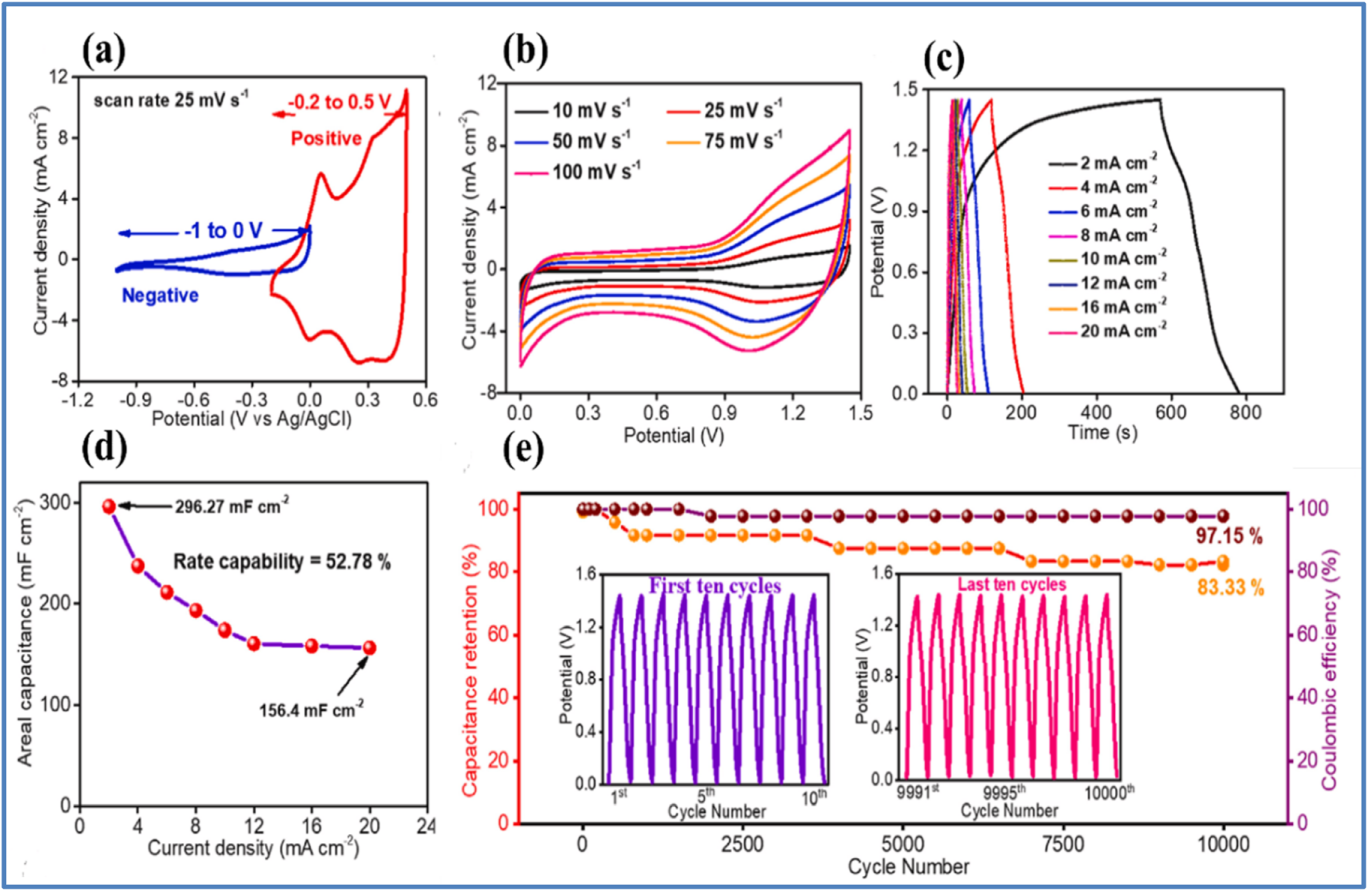
(a) CV curves of the CoTe@C–NiF hybrid electrode in the negative and positive windows at 25 mV s^−1^ (b and c) CV and GCD curves at different *ν* and *I*_d_, respectively (d) *C*_a_*vs. I*_d_ curve (e) cyclic stability curve along with the first and last ten GCD cycles (inset) of the CoTe@C–NiF//CoTe@C–NiF symmetric device (reproduced from ref. 28 with permission from Elsevier© 2021).

Kshetri *et al.*^28^ developed a metal–organic framework (MOF)-derived cobalt telluride-carbon composite on nickel foam (CoTe@C–NiF) as an electrode for SCs. Fig. 3 depicts the schematic representation and structural characterizations of CoTe@C–NiF electrode. The CoTe@C–NiF hybrid material exhibits a unique feature as a bifunctional electrode, functioning in different potential windows. In a three-electrode system, it can serve as a working electrode with potential ranges of −0.8 to 0.0 V for NE and 0.0 to 0.5 V for PE. The electrochemical characterization of CoTe@C–NiF electrode is given in Fig. 4. In the negative potential region, the CoTe@C–NiF electrode demonstrates a maximum areal capacitance (*C*_a_) of 307.5 mF cm^−2^ at a *I*_d_ of 1 mA cm^−2^, retaining 162.0 mF cm^−2^ at 20 mA cm^−2^, which indicates a rate capability of 52.03%. Conversely, in the positive potential region, the electrode achieves *C*_a_ of 1038 mF cm^−2^ and 920 mF cm^−2^ at the same *I*_d_, respectively, resulting in an 88.63% rate capability. Additionally, the CoTe@C–NiF material demonstrates excellent long-term electrochemical stability during CD cycling in both potential windows. In the negative window, the material retains 86.78% of its initial *C*_a_ and 98.57% *η* after 10 000 cycles. In the positive window, it retains 87.50% of its *C*_a_ and 98.97% *η* under the same conditions. For practical application, two identical CoTe@C–NiF hybrid electrodes were utilized as both NE and PE in a SC device with 2 M KOH electrolyte and cellulose filter paper as a separator. The device (Fig. 5) exhibited *C*_a_ of 296.27 mF cm^−2^ at 2 mA cm^−2^ and 156.4 mF cm^−2^ at 20 mA cm^−2^, demonstrating a rate capability of 52.78%. The device also showed remarkable electrochemical stability, with 83.33% retention of *C*_s_ and 97.15% *η* after 10 000 cycles. The maximum *E*_d_ achieved was 43.84 W h kg^−1^ at a *P*_d_ of 738.88 W kg^−1^, with an *E*_d_ of 21.95 W h kg^−1^ even at a high *P*_d_ of 6173.44 W kg^−1^.

In coordination chemistry, phosphorus, a multivalent non-metallic in the nitrogen group, plays an essential role as a donor atom. Phosphoric compounds are found in a variety of forms, that mainly includes metal phosphates and metal phosphides. The potential of these compounds to improve the performance of electrodes used in energy storage applications such as catalysis, SCs, and LIBs makes them interesting. Particularly, transition metal phosphides are regarded as potential SCs electrode materials due to their metalloid properties, strong electrochemical activity, and outstanding electrical conductivity. The advantages of phosphides over oxides stem from phosphorus's lower electronegativity (2.19 on the Pauling scale) compared to oxygen (3.44), and its larger atomic radius (0.109 nm for phosphorus *versus* 0.074 nm for oxygen), which contribute to distinct physicochemical properties.^29^ In metal phosphides, the phosphorus atoms attract valence electron density due to the ionic nature of these compounds. As the phosphorus content increases, the degree of electron delocalization decreases, leading to a reduction in ionic character. Consequently, metal-rich phosphides, such as M_3_P, M_2_P, and MP, contain more free electrons, which enhance electrical conductivity.^30^

The term “phosphate” encompasses oxyanions of pentavalent phosphorus, ranging from simple PO_4_^3−^ ions to complex ring and chain structures, and even extending to infinite networks. This variety in structural arrangements, along with the numerous cations they can coordinate with and the inclusion of additional anions or molecules, results in a wide diversity of metal phosphates.^30^ Transition metal phosphates or pyrophosphates (TMPs) are particularly advantageous as SCs electrode materials due to their remarkable attributes, including reversible redox chemistry, a robust open framework, mesoporous architecture, exceptional electrochemical and thermal stability, abundant natural resources, and cost-effectiveness. The mesoporous morphology of TMPs provides ample interstitial space for facile ion diffusion, thereby enhancing electrochemical performance. The interplay of metal cations and the strong covalent P–O bonds within TMPs impart exceptional chemical resilience and augmented electrical conductivity. Moreover, the open framework of TMPs facilitates unhindered electrolyte penetration to active sites, fostering expedited ion transport and optimized charge transfer kinetics. The availability of oxygen within TMPs extends their operational potential window, mitigating degradation and consequently elevating energy storage capacity. These collective advantages render TMPs highly promising candidates for advanced energy storage applications.^31,32^

An *et al.*^33^ synthesized Ni_2_P nanoparticles on reduced graphene oxide (rGO) using a low-temperature solid-state reaction method. They studied these materials as electrochemical pseudocapacitors. The Ni_2_P/rGO composite exhibited a *C*_s_ of 2354 F g^−1^ at 1 mA cm^−2^, significantly higher than the 1597 F g^−1^ achieved by the Ni_2_P nanoparticles alone. The enhanced *C*_s_ of the Ni_2_P/rGO composite can be attributed to several factors. First, the precise assembly of Ni_2_P nanoparticles with graphene oxide nanosheets, followed by *in situ* reduction, aids in creating hybrid materials with molecular-level dispersion and strong interfacial interactions. Second, the close integration of nickel phosphide nanoparticles with conductive graphene facilitates efficient charge transport, enhancing overall electronic conductivity. Third, incorporating Ni_2_P nanoparticles onto the mechanically robust rGO prevents the aggregation of electroactive material, reduces the restacking of graphene sheets, and increases the electrochemically active surface area, thus maximizing the benefits of Ni_2_P pseudocapacitance and graphene-based double-layer capacitance.

**Fig. 6:**
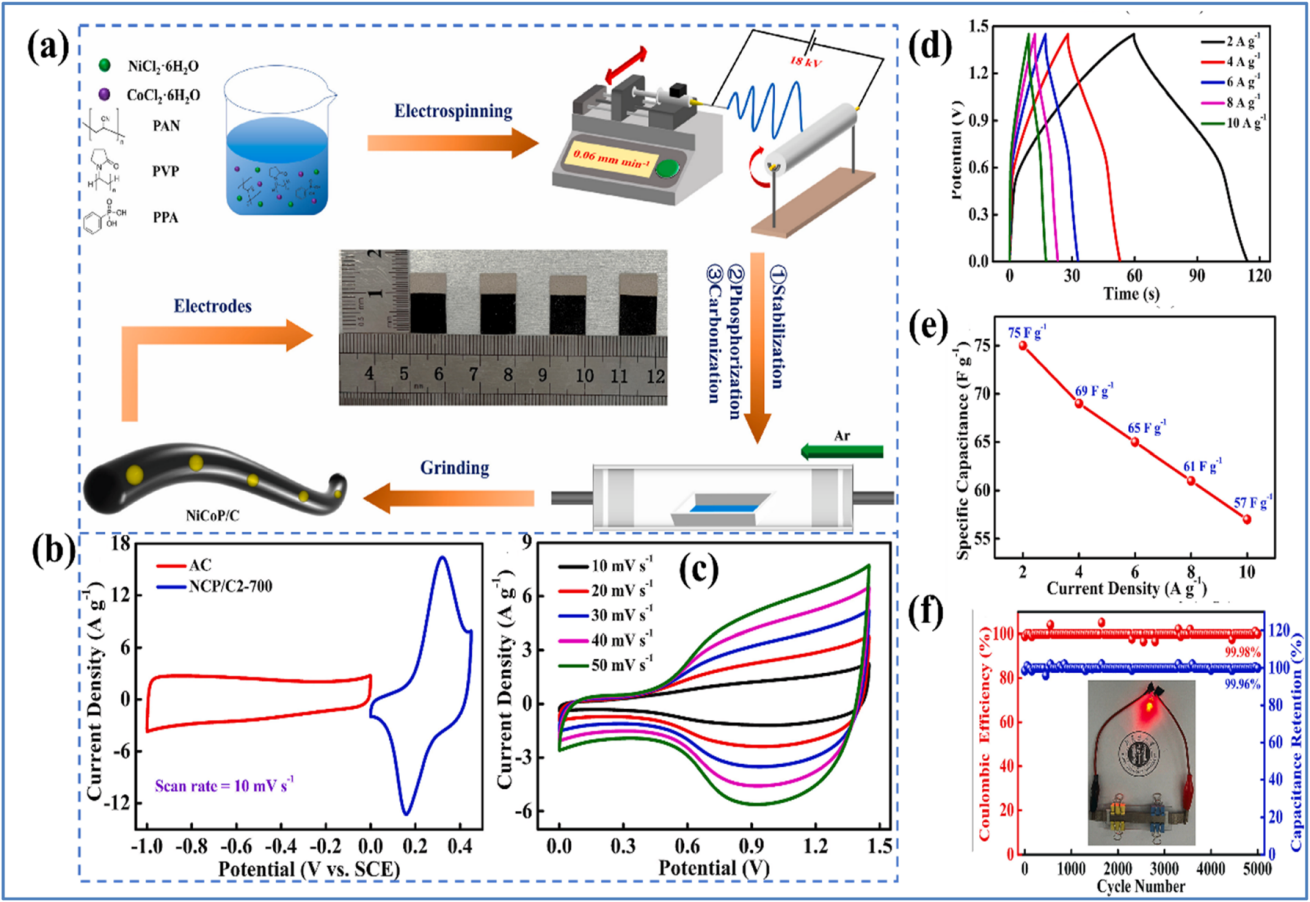
(a) Schematic representation of the preparation of NiCoP/C composite (b) CV curves of NCP/C2-700 and AC at a *ν* of 10 mV s^−1^ (c) CV curves at different *ν* (d) GCD curves (e) rate capability curve of NCP/C2-700//AC device (f) cycling life and *η* of NCP/C2-700//AC at 10 A g^−1^ and a digital photo of a red LED illuminated by NCP/C2-700//AC device (reproduced from ref. 34 with permission from Elsevier© 2024).

Zhao *et al.*^34^ fabricated nickel cobalt phosphide/carbon nanofibers (NiCoP/C2-700) with an approximate diameter of 200 nm through a process combining electrospinning and calcination. The synthesis process and electrochemical characterization is illustrated in Fig. 6.

**Fig. 7:**
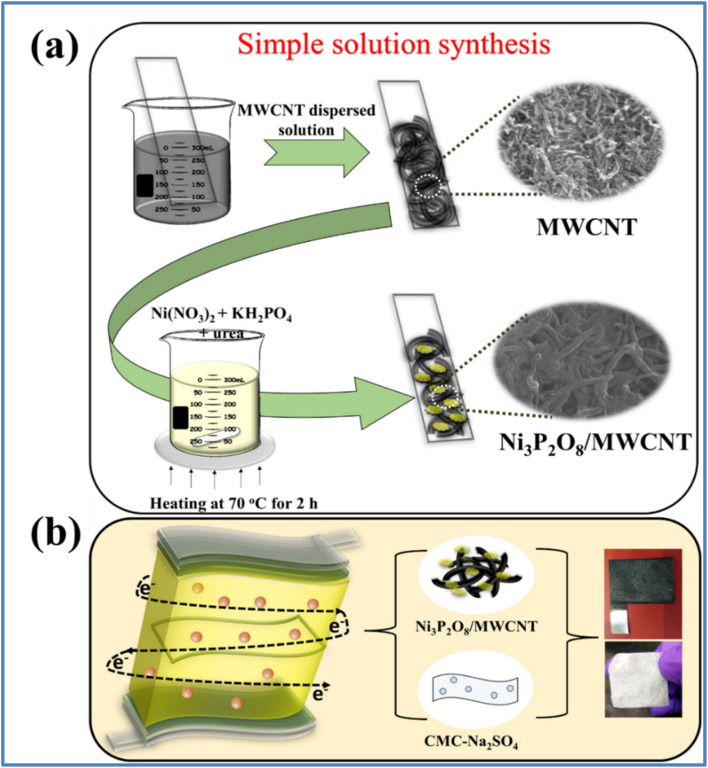
(a) Schematic showing easy solution chemistry for coupling hierarchically constructed Ni_3_P_2_O_8_/MWCNT on SS substrate (b) schematic representation of Ni_3_P_2_O_8_/MWCNT solid-state SSC device assembly with CMC-Na_2_SO_4_ electrolyte membrane (reproduced from ref. 35 with permission from Elsevier© 2023).

The resulting NiCoP/C2-700 material demonstrated a *C*_s_ of 478 F g^−1^ at 2 A g^−1^ in a 3 M KOH electrolyte. Impressively, it retained 99.99% of its initial *C*_s_ even after 5000 charge/discharge cycles at 10 A g^−1^, highlighting its excellent electrochemical properties. Additionally, an ASC was engineered using NiCoP/C2-700 as the PE and AC as the NE. This device achieved an *E*_d_ of 16.72 W h kg^−1^ at a high *P*_d_ of 7250 W kg^−1^. Notably, the capacitance loss after 5000 cycles at 10 A g^−1^ was only 0.04%. This remarkable stability is largely due to the role of carbon nanofibers as a supporting structure, which enhances the stability of the NiCoP nanoparticles. The outstanding electrochemical performance is attributed to the synergistic interaction between the NiCoP nanoparticles and the carbon nanofibers. Agarwal *et al.*^35^ developed a composite of Ni_3_P_2_O_8_ nanodots anchored on multiwalled carbon nanotubes (Ni_3_P_2_O_8_/MWCNT) using a dip-and-dry method followed by chemical bath deposition as illustrated in Fig. 7(a). The corresponding flexible all-solid-state SSC (Fig. 7(b)), utilizing a carboxymethyl cellulose-Na_2_SO_4_ (CMC-Na_2_SO_4_) neutral gel electrolyte.

**Fig. 8:**
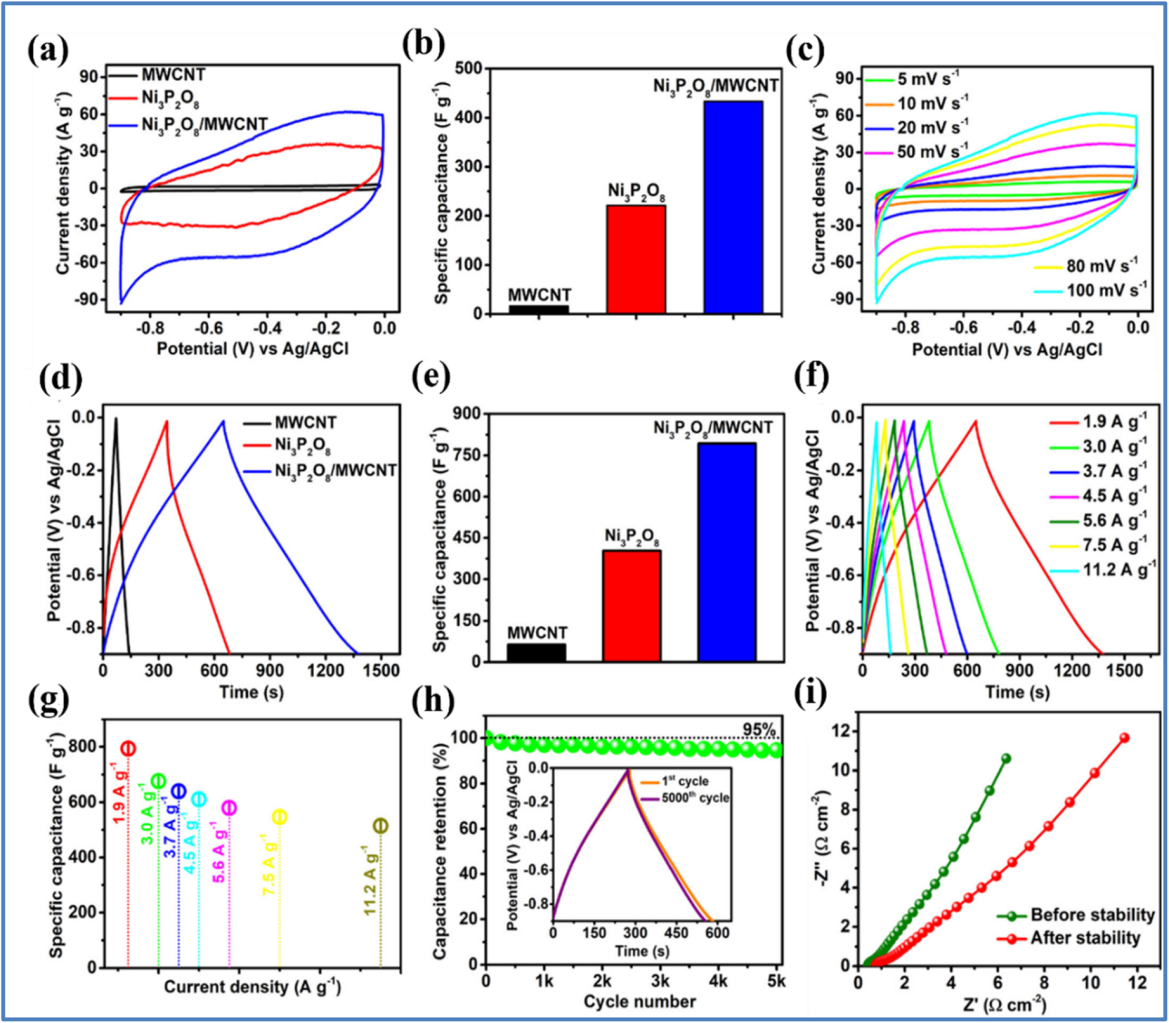
(a) CV curves and (b) corresponding *C*_s_ plot for MWCNT, Ni_3_P_2_O_8_, and Ni_3_P_2_O_8_/MWCNT at 100 mV s^−1^ (c) CV analysis of the prepared Ni_3_P_2_O_8_/MWCNT composite electrode: at different *ν* between 5 and 10 mV s^−1^ (d) GCD curves and (e) corresponding *C*_s_ plot for MWCNT, Ni_3_P_2_O_8_, and Ni_3_P_2_O_8_/MWCNT at 1.9 A g^−1^ (f) GCD measurement of Ni_3_P_2_O_8_/MWCNT composite at different *I*_d_ (g) corresponding *C*_s_ values, (h) cyclic retention plot (inset shows GCD curves for the first and last cycle) and (i) Nyquist plot of Ni_3_P_2_O_8_/MWCNT before and after stability (reproduced from ref. 35 with permission from Elsevier© 2023).

The electrochemical studies of MWCNT, Ni_3_P_2_O_8_, and Ni_3_P_2_O_8_/MWCNT composite electrodes are given in Fig. 8. The high redox activity of Ni_3_P_2_O_8_ combined with the large surface area provided by the robust carbon framework resulted in a significant synergistic effect within the composite. This synergy led to an increased CV enclosed area and greater redox current compared to the individual components, indicating an improved energy storage capability. The MWCNTs in the composite help reduce the resistance of the host material and enhance the charge collection efficiency, facilitating ion transport within the electrode. Consequently, the Ni_3_P_2_O_8_/MWCNT composite exhibits a significantly higher *C*_s_ of 793.1 F g^−1^ at 1.9 A g^−1^ and maintains 64.9% (514.6 F g^−1^) of its rate capability even at 11.2 A g^−1^. The composite retains more than 95% of its *C*_s_ after 5000 GCD cycles, demonstrating high cycling stability. Ni_3_P_2_O_8_/MWCNT solid-state SSC device assembly with CMC-Na_2_SO_4_ electrolyte membrane achieved maximum specific energy of 72.3 W h kg^−1^ and a *P*_d_ of 6.4 kW kg^−1^ within an extended voltage window of 1.8 V. Additionally, the device exhibits excellent deformation tolerance, retaining 103% of its performance under a mechanical bending angle of 170°.

Research into supercapacitors has generated a lot of interest in MXenes. MXene–carbon-based hybrid materials have attracted significant attention in the field of supercapacitors due to their unique combination of properties.

**Fig. 9:**
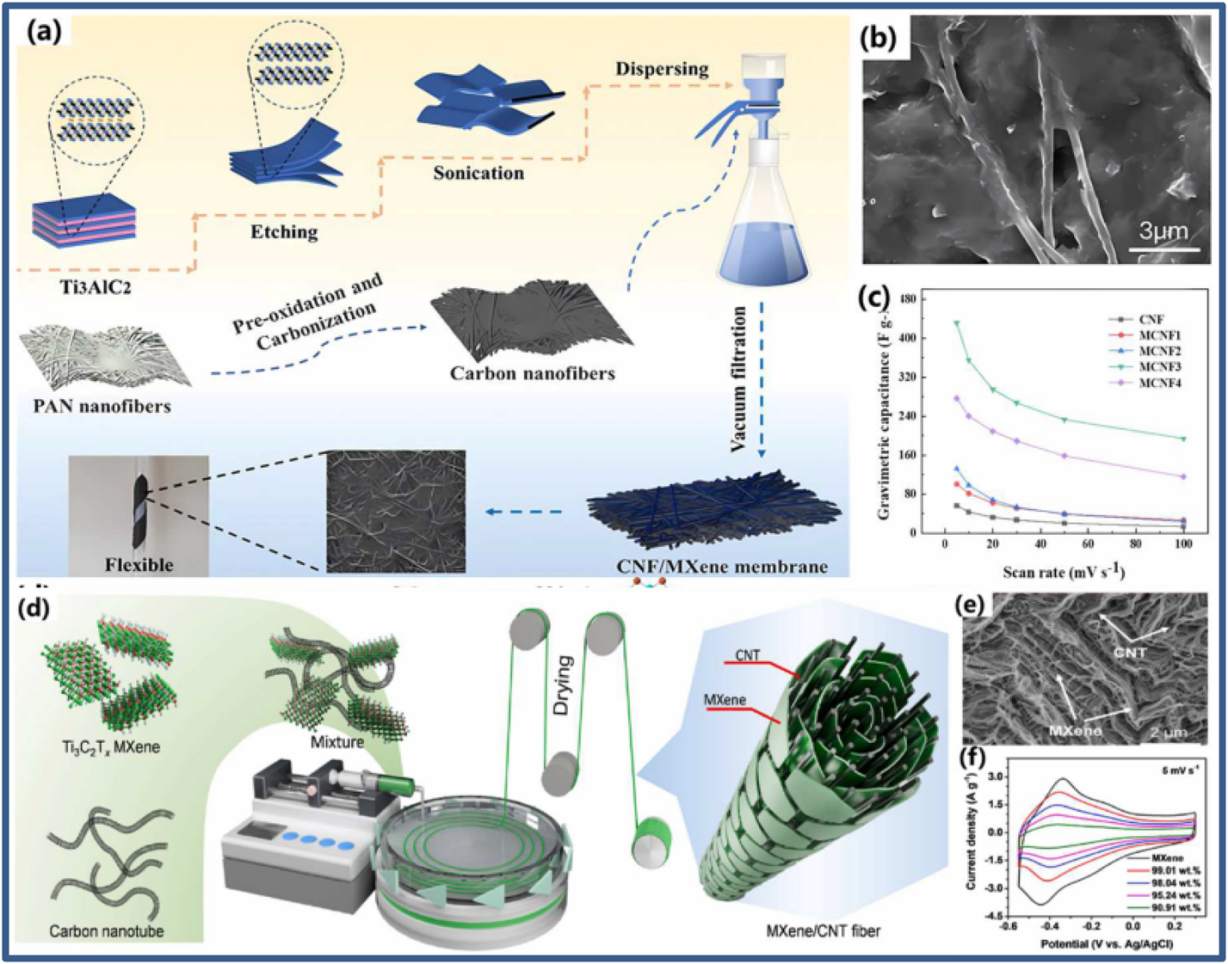
One-dimensional carbon/MXene composites: (a) schematic diagram, (b) SEM, and (c) capacitance value of MXene/N-CNT (d) schematic diagram and (e) TEM of MXene/CNT fiber, (f) CV curves (reproduced from ref. 36 with permission from American Chemical Society© 2023).

Wang *et al.*^36^ developed MXene/N-CNT composite materials with a hierarchical porous structure that effectively inhibited the self-aggregation of 2D MXene and 1D N-CNT, as demonstrated in Fig. 9(a)–(c).^36^ This structure enhances the interlayer spacing of MXene and increases the specific surface area of the composite, providing more active sites for reactions and ion diffusion channels. As a result, the composite material exhibits a significantly improved specific capacitance (167.2 F g^−1^) compared to the pure MXene electrode. Furthermore, the asymmetric supercapacitor device based on these composite shows a high capacitance retention rate of 73.2% after 10 000 cycles, a high coulombic efficiency of 97.5%, and a maximum energy density of 12.1 W h kg^−1^. Similarly, Chen *et al.*^37^ fabricated a Ti_3_C_2_T_*x*_ MXene/CNTs composite for supercapacitor applications, demonstrating that the synthesized material achieved a high capacitance of 300 F g^−1^ at a current density of 1 A g^−1^ and exhibited excellent rate performance of 199 F g^−1^ even at a current density of 500 A g^−1^. Additionally, the material maintained 92% of its capacitance after 10 000 cycles at a high current density of 20 A g^−1^, highlighting its impressive long-term cycle stability. The incorporation of CNTs into MXene successfully prevented self-restacking, facilitated rapid ion diffusion, and ensured excellent high-rate performance.

**Fig. 10:**
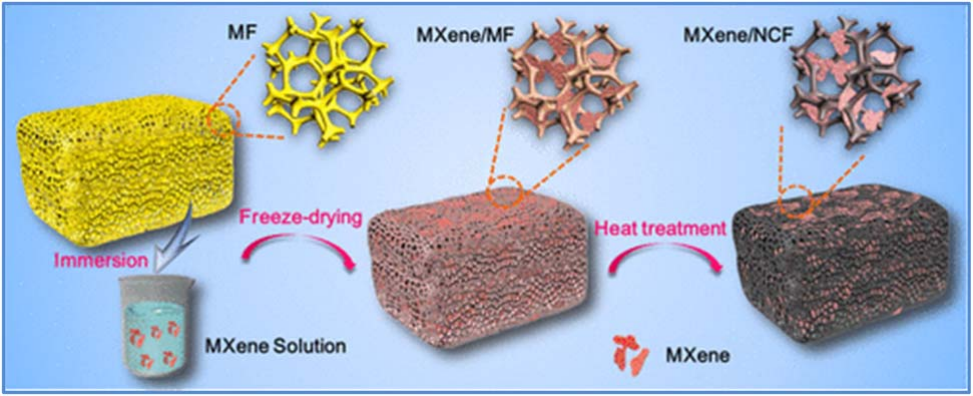
MXene/N-doped carbon foam (NCF) composite with three-dimensional (3D) hollow interconnected neuron-like architecture (reproduced from ref. 39 with permission from American Chemical Society© 2020).

Zhao *et al.*^38^ developed a multifunctional Ti_3_C_2_T_*x*_ MXene/carbon nanotube (MXene/CNT) hybrid fiber using a wet spinning method, as shown in Fig. 9(d)–(f).^38^ With approximately 1 wt% CNT content, the hybrid fiber achieved a high tensile strength of 61 ± 7 MPa, a conductivity of 1142.08 ± 40.04 S cm^−1^, and an impressive specific capacitance of around 295 F g^−1^. When the CNT content was increased to 9 wt%, the fiber exhibited a maximum strain of 161 ± 19 MPa and further enhanced conductivity (1715 ± 22 S cm^−1^). These MXene/CNT fibers possess excellent mechanical properties, making them suitable for weaving into energy storage textiles, with a maximum energy density of approximately 6.08 mW h cm^−3^. Similarly, Sun *et al.*^39^ developed a novel MXene/N-doped carbon foam (MXene/NCF) compressible composite with a 3D hollow interconnected structure shown in Fig. 10. The NCF provides additional pseudocapacitance through nitrogen atom doping, while supporting MXene nanosheets to form stable 3D interconnected frameworks that offer efficient ion diffusion and electron transport pathways. The MXene enhances the composite's conductivity and hydrophilicity. Due to the synergistic effects between MXene and NCF, the composite material exhibited outstanding capacitance performance of 332 F g^−1^ (3162 mF cm^−3^), 64% rate performance (0.5–100 A g^−1^), and excellent capacity retention of 99.2% after 10 000 cycles. Moreover, the material maintains stable electrochemical properties and morphology even under repeated 60% strain (Table 1).

**Table 1:** Comparative table of carbon-based binary TMC and their performance

S. no.	Composite material	Binary TMC	Conductivity	Current density	Cyclic stability	References
1	Activated carbon nanofibers/cobalt ferrite	CNF/CoFe_2_O_4_	188.36 F g^−1^	0.5 A g^−1^	80% after 10 000 cycles	17
2	Ultra-fine ruthenium quantum dots on a reduced graphene oxide	RuO_2_/rGO	1120 F g^−1^	1 A g^−1^	89% after 10 000 cycles	18
3	Bimetallic hydroxide/multi-walled carbon nanotube	La–NiCo LDH/MWCNT	4396 F g^−1^	1 A g^−1^	70.31% after 3000 cycles	19
4	Sulfide-based carbon composite electrode	CFS/CNT	667 F g^−1^	15 A g^−1^	100% after 5000 cycles	25
5	Hexagonal VS_2_ nanoparticles on a multi-walled carbon nanotube	MWCNT	830 F g^−1^	2 mV s^−1^	95.9% after 10 000 cycles	26
6	Nickel selenide nanoparticles on graphene nanosheets	NiSe–G	1280 F g^−1^	1 A g^−1^	98% after 2500 cycles	27
7	Cobalt telluride–carbon composite on nickel foam	CoTe@C–NiF	1038 F g^−1^	2 mA cm^−2^	97.15% after 10 000 cycles	28
8	Ni_2_P nanoparticles on reduced graphene oxide (rGO)	Ni_2_P/rGO	2354 F g^−1^	1 mA cm^−2^	94% after 5000 cycles	33
9	Nickel cobalt phosphide/carbon nanofibers	NiCoP/C2-700	478 F g^−1^	2 A g^−1^	99.99% after 5000 cycles	34
10	Ni_3_P_2_O_8_ nanodots anchored on multiwalled carbon nanotubes	Ni_3_P_2_O_8_/MWCNT	793.1 F g^−1^	1.9 A g^−1^	95% after 5000 cycles	35
11	MXene/CNTs composite materials	MXene/N-CNT	167.2 F g^−1^	1 A g^−1^	73.2% after 10 000 cycles	36
12	MXene/CNTs composite	Ti_3_C_2_T_*x*_ MXene/CNTs	300 F g^−1^	1 A g^−1^	92% after 10 000 cycles	37
13	Multifunctional MXene/carbon hybrid fibers	Ti_3_C_2_T_*x*_ MXene/CNTs	295 F g^−1^	1 A g^−1^	95% after 5000 cycles	38
14	Novel MXene/N-doped carbon foam	MXene/NCF	332 F g^−1^	0.5–100 A g^−1^	99.2% after 10 000 cycles	39
15	Delaminated Mxene heterostructure film	Ti_3_C_2_T_*x*_/g-C_3_N_4_	414 F g^−1^	1 A g^−1^	94.93% after 2500 cycles	40

### 2.2 Conducting polymer-based binary TMC

Conducting polymers (CPs), such as poly(3,4-ethylenedioxythiophene) (PEDOT), polypyrrole (PPy), polythiophene (PTH), polyacetylene (PA), and polyaniline (PANI), have garnered significant interest since their discovery in the 1960s for applications in sensors, electrochromic devices, and energy storage. These materials are noted for their high electrical conductivity and excellent capacitive properties. Their simple chemical composition—consisting mainly of carbon, hydrogen, nitrogen, or sulphur also suggests a cost-effective production.^41^ Devices based on conducting polymers exhibit higher *C*_s_ compared to EDLCs and demonstrate faster kinetics than most inorganic batteries, offering a promising solution to bridge the gap between these two technologies in energy storage applications. However, the reduction–oxidation processes in conducting polymers lead to mechanical stress, limiting their stability over numerous CD cycles. This repeated swelling and shrinking during cycling compromises their long-term stability and performance. Consequently, using conducting polymers alone as SC electrodes is not ideal. To address these challenges, hybrids that combine conducting polymers with transition metal compounds or carbon materials are popular. These hybrids take advantage of the complementary properties of each component, leading to higher *C*_s_ and improved stability.^7,41,42^

**Fig. 11:**
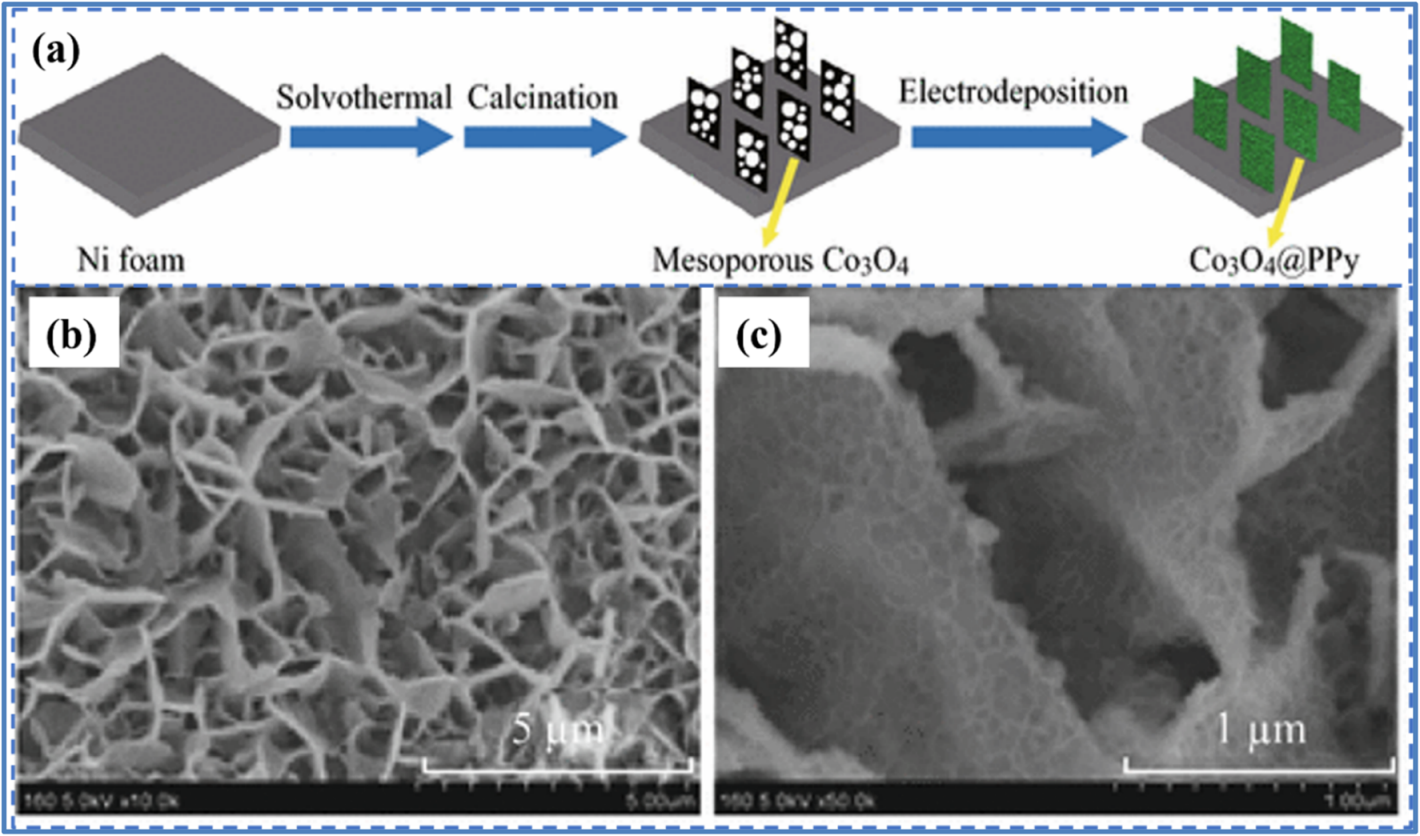
(a) Schematic diagram for the synthesis of mesoporous Co_3_O_4_@PPy hybrid nanosheet arrays (b) and (c) SEM images of Co_3_O_4_@PPy hybrid at lower and higher resolution (reproduced from ref. 43 with permission from Springer© 2016).

**Fig. 12:**
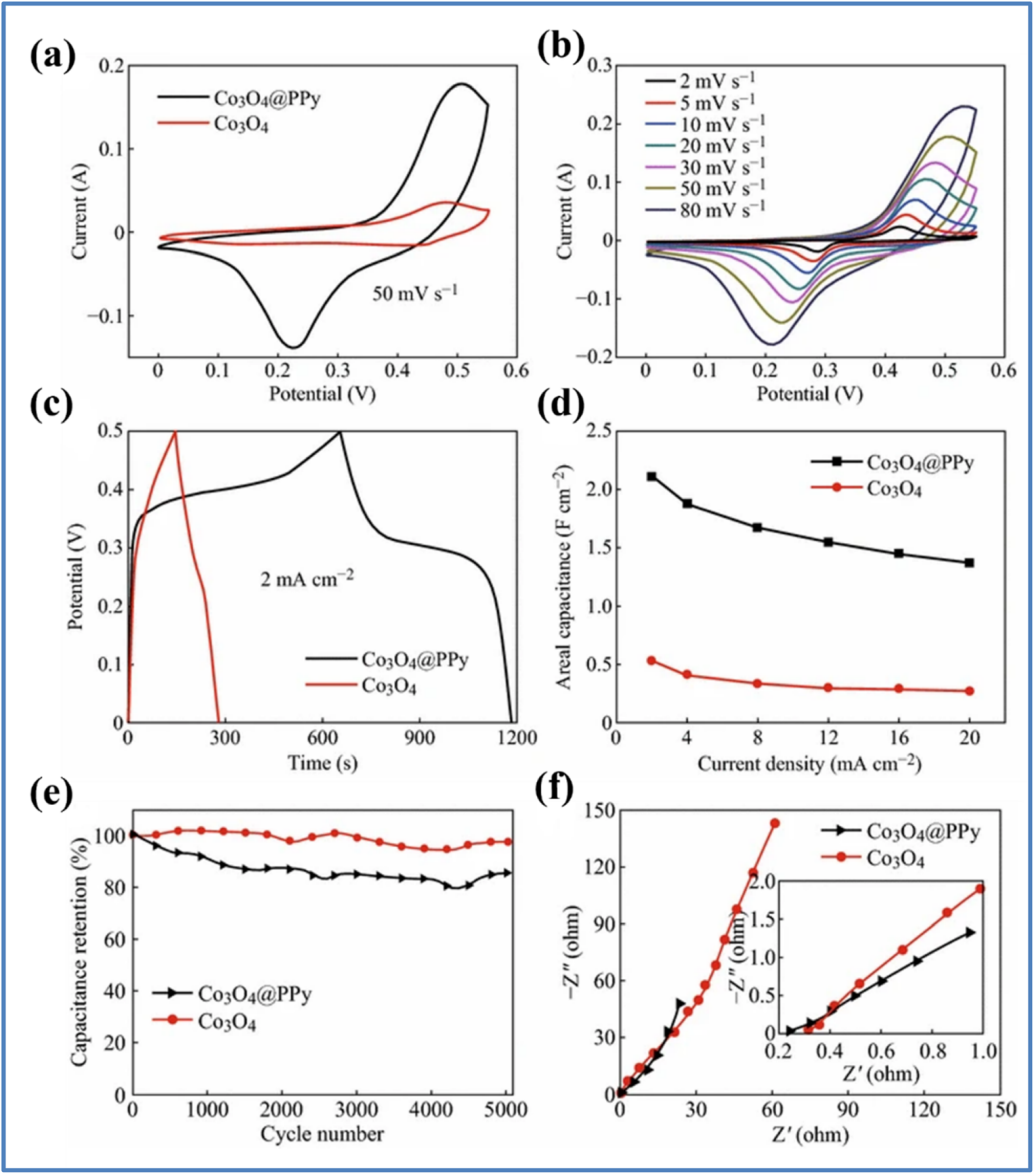
(a) CV curves of the Co_3_O_4_@ppy and Co_3_O_4_ electrode at a 50 mV s^−1^ (b) CV curves of the Co_3_O_4_@ppy and Co_3_O_4_ electrode at different *ν*. (c) GCD curves of the Co_3_O_4_@ppy hybrid electrode and Co_3_O_4_ electrode at 2 mA cm^−2^ (d) *C*_a_ of the Co_3_O_4_@ppy and Co_3_O_4_ electrodes at varying *I*_d_ (e) cycling stability of the Co_3_O_4_@ppy and Co_3_O_4_ electrodes at a *ν* of 50 mV s^−1^ for 5000 cycles (f) EIS curves of the Co_3_O_4_@ppy and Co_3_O_4_ electrodes (reproduced from ref. 43 with permission from Springer© 2016).

Yang *et al.*^43^ synthesized Co_3_O_4_@PPy core/shell nanosheet arrays using a combination of solvothermal synthesis and electrodeposition techniques. They grew a thin layer of polypyrrole (PPy) on the surface of mesoporous Co_3_O_4_ nanosheet arrays through electrodeposition. The process of fabrication of Co_3_O_4_@PPy hybrid along with its SEM images is given in Fig. 11. The electrochemical study was performed in 1 M KOH as displayed in Fig. 12. The hybrid Co_3_O_4_@PPy electrode, after an 8 minute electrodeposition process, exhibited the highest *C*_a_ of 2.11 F cm^−2^ at a *I*_d_ of 2 mA cm^−2^, which is significantly higher than the 0.54 F cm^−2^ achieved by the pristine Co_3_O_4_ electrode. Even when the *I*_d_ was increased to 20 mA cm^−2^, the Co_3_O_4_@PPy electrode maintained an *C*_a_ of 1.37 F cm^−2^, indicating excellent rate capability (65%). In contrast, the pristine Co_3_O_4_ electrode only retained 50% of its *C*_a_ at the same *I*_d_. Notably, after coating with a PPy layer, the CV curve of the Co_3_O_4_@PPy electrode expanded significantly, demonstrating a substantial increase in *C*_a_ due to the synergistic effects of Co_3_O_4_ and PPy. The PPy layer enhances electrical conductivity, improving electron transport through the nanosheets. Additionally, PPy contributes to extra pseudocapacitance through ion doping/dedoping in an alkaline solution. The Co_3_O_4_@PPy hybrid electrode retained 85.5% of its initial *C*_a_ after 5000 cycles, indicating superior cycling stability. In comparison, the pristine Co_3_O_4_ electrode retained 97.7% of its *C*_a_, showing a 12% decrease in the hybrid electrode's retention. This decline is attributed to the inherent poor cycling stability of PPy, which undergoes significant volumetric changes during ion doping/dedoping. The Nyquist plots at high frequencies revealed that the equivalent series resistance (ESR) of the Co_3_O_4_@PPy hybrid electrode (0.238 Ω) was lower than that of the pristine Co_3_O_4_ electrode (0.319 Ω), indicating improved electrical conductivity due to the PPy coating.

Kong *et al.*^44^ developed NiCo_2_O_4_ nanowire arrays coated with polypyrrole, referred to as NiCo_2_O_4_@PPy NWAs. These hybrid electrodes achieved a *C*_s_ of 2244.5 F g^−1^ at a *I*_d_ of 1 A g^−1^, significantly surpassing the 1189.4 F g^−1^ of the pure NiCo_2_O_4_ NWAs electrode. Even at an increased *I*_d_ of 30 A g^−1^, the NiCo_2_O_4_@PPy NWAs maintained a *C*_s_ of 1358 F g^−1^, showcasing a notable rate capability of approximately 60.5%. In comparison, the pure NiCo_2_O_4_ NWAs only retained about 52.6% of their *C*_s_ at the same *I*_d_. The hybrid NiCo_2_O_4_@PPy NWAs also demonstrated strong cycling stability, retaining 89.5% of their initial *C*_s_ after 5000 cycles and 82.9% after 10 000 cycles. Throughout extended cycling, the *η* remained mostly above 97%, indicating efficient electron transfer for charge storage and release. However, the pure NiCo_2_O_4_ NWAs exhibited a greater *C*_s_ loss of around 10.9% after 5000 cycles. The primary cause of this decay in NiCo_2_O_4_ is attributed to its dissolution in the electrolyte, whereas the *C*_s_ loss in the conducting polymers is due to structural defects from repeated swelling and shrinking of polymer chains. The improved cycling stability of the NiCo_2_O_4_@PPy NWAs is due to their coaxial structure, where the NiCo_2_O_4_ core provides a robust framework that interconnects with the PPy nanospheres, while the PPy layer prevents the dissolution of NiCo_2_O_4_ in the electrolyte. The charge transfer resistance (*R*_ct_) of the hybrid NiCo_2_O_4_@PPy NWAs electrode is approximately 8.8 Ω, lower than the 13.7 Ω of the pristine NiCo_2_O_4_ NWAs electrode. After 10 000 cycles, the *R*_ct_ only slightly increased from 8.8 to 11.2 Ω, suggesting that the long-term CD process did not significantly damage the NiCo_2_O_4_@PPy hybrid electrode. Furthermore, a flexible ASC device was successfully constructed using the NiCo_2_O_4_@PPy NWAs as one electrode and AC as the other. This device achieved a high *E*_d_ of 58.8 W h kg^−1^ at a *P*_d_ of 365 W kg^−1^, outstanding *P*_d_ of 10.2 kW kg^−1^ at an *E*_d_ of 28.4 W h kg^−1^, and excellent cycling stability with approximately 89.2% *C*_s_ retention after 5000 cycles. The flexible nature of the device makes it highly suitable for future portable and wearable electronic devices. The innovative three-dimensional coaxial architecture design paves the way for developing high-performance, flexible SCs.

**Fig. 13:**
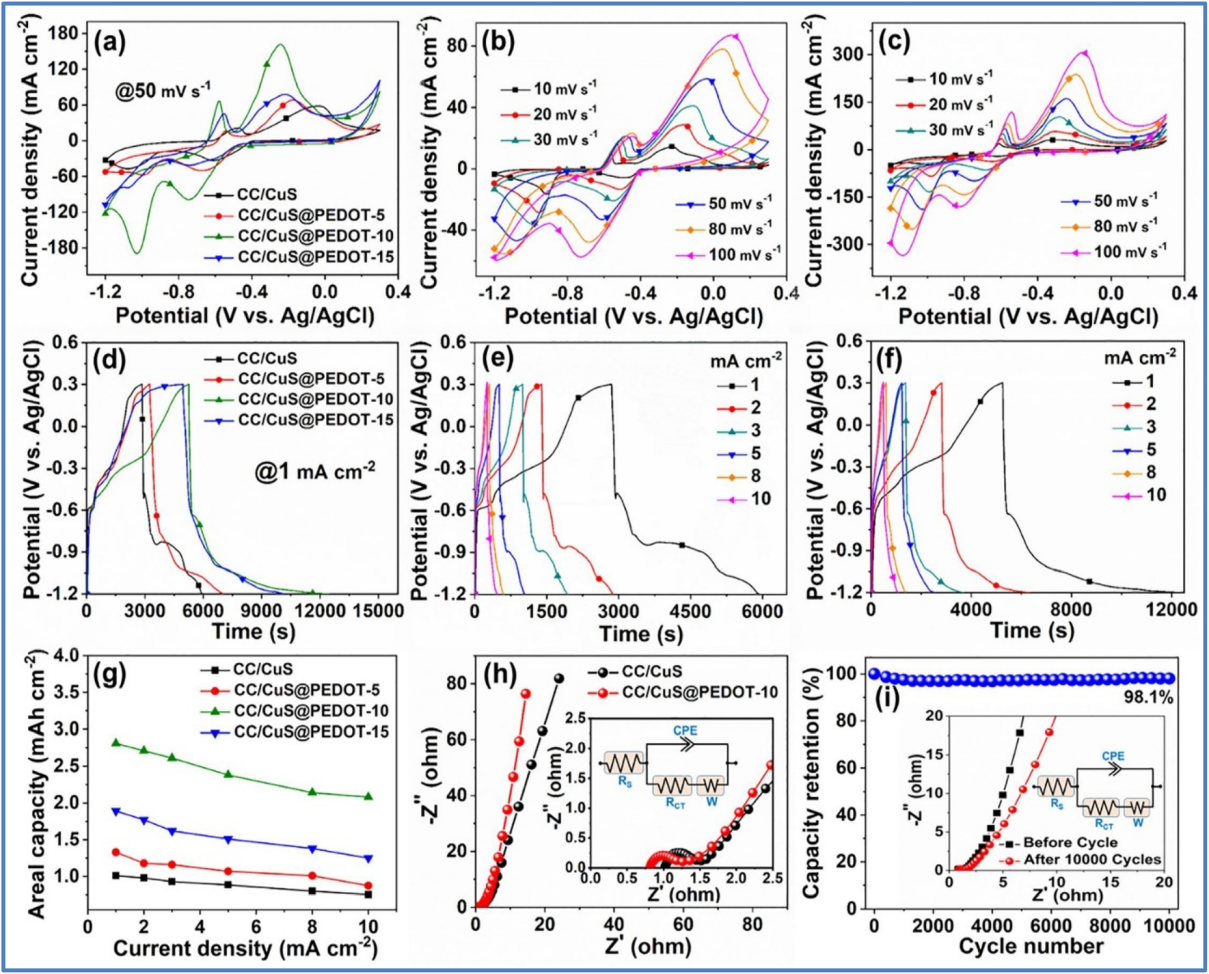
(a) Comparative CV curves of CC/CuS, CC/CuS@PEDOT-5, CC/CuS@PEDOT-10 and CC/CuS@PEDOT-15 at 50 mV s^−1^, CV curves of (b) CC/CuS nanoplate arrays and (c) CC/CuS@PEDOT-10 hybrid at different *ν* (d) comparative GCD profiles of CC/CuS, CC/CuS@PEDOT-5, CC/CuS@PEDOT-10 and CC/CuS@PEDOT-15 at 1 mA cm^−2^, GCD curves of (e) CC/CuS nanoplate arrays and (f) CC/CuS@PEDOT-10 hybrid at various *I*_d_, (g) plot of *C*_a_ values *versus* the *I*_d_ for CC/CuS, CC/CuS@PEDOT-5, CC/CuS@PEDOT-10 and CC/CuS@PEDOT-15, (h) Nyquist plots of CC/CuS nanoplate arrays and CC/CuS@PEDOT-10 hybrid (inset shows the magnified Nyquist plots in the higher frequency region and the equivalent circuit), and (i) cycling performance of the CC/CuS@PEDOT-10 hybrid NE for 10 000 consecutive GCD cycles at 5 mA cm^−2^ (inset represents the Nyquist plots before and after the cycling with the fitted equivalent circuit) (reproduced from ref. 45 with permission from Elsevier© 2022).

Das *et al.*^45^ developed a hybrid NE by combining solvothermal and potentiostatic electrodeposition techniques to deposit CuS@PEDOT-*x* (*x* = 5, 10, 15) onto a carbon cloth (CC) substrate. The electrochemical analysis of these electrodes is given in Fig. 13. This CC/CuS@PEDOT-10 electrode achieved the highest *C*_s_ of 2.81 mA h cm^−2^ at a *I*_d_ of 1 mA cm^−2^, outperforming the CC/CuS electrode, which reached around 1.01 mA h cm^−2^ under the same conditions.

**Fig. 14:**
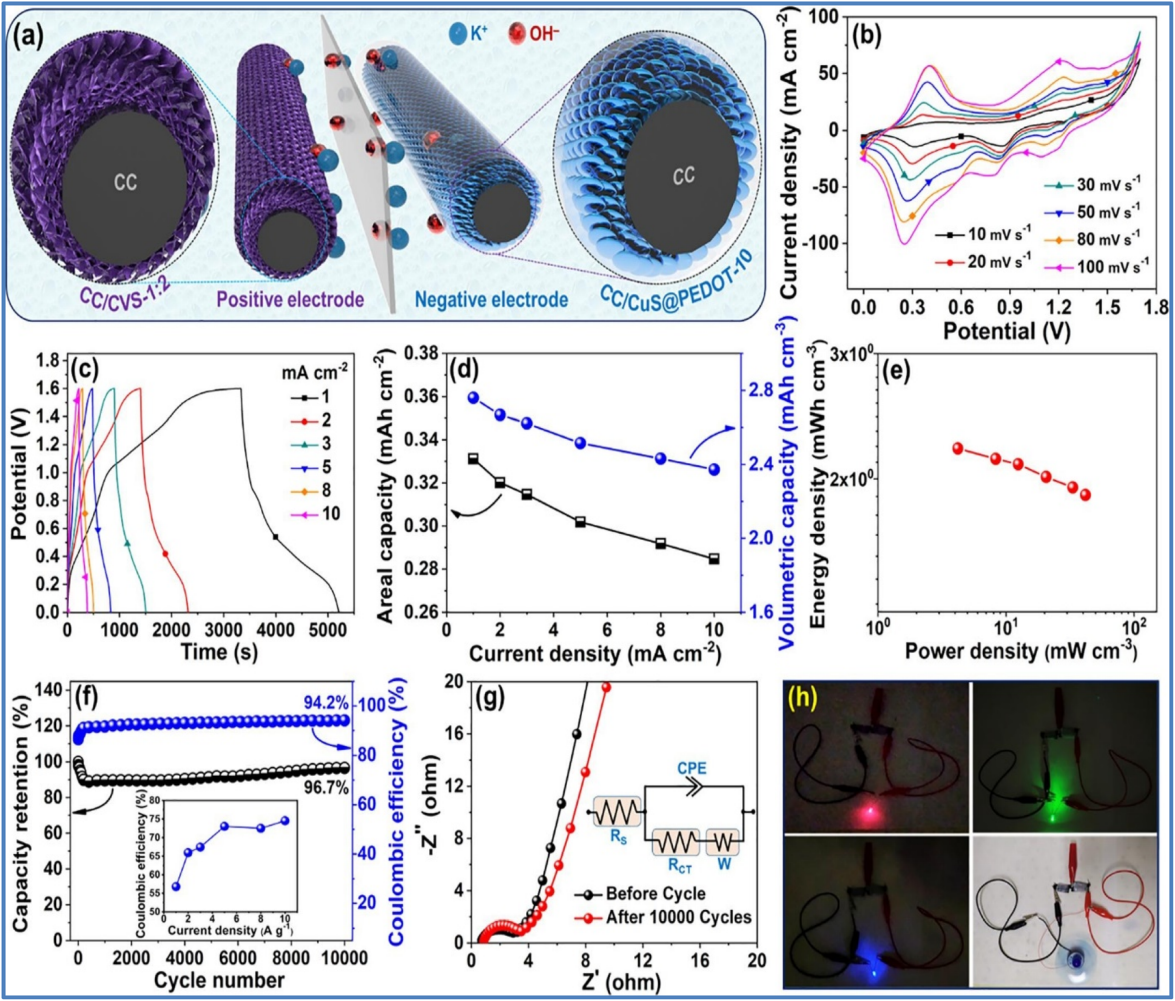
(a) Schematic of CC/CVS-1:2//CC/CuS@PEDOT-10 asymmetric device, electrochemical properties of CC/CVS-1:2//CC/CuS@PEDOT-10 asymmetric device (b) CV at various *ν* (c) GCD at various *I*_d_ (d) areal capacity *vs. I*_d_ (e) Ragone plot (f) capacity retention and *η* over 10 000 cycles (g) EIS plot before and after cycling with fitted equivalent circuit, and (h) illuminating red, green, and blue LEDs and powering a small electrical motor fan using two serially connected CC/CVS-1:2//CC/CuS@PEDOT-10 asymmetric device (reproduced from ref. 45 with permission from Elsevier© 2022).

The hybrid electrode demonstrated excellent rate capability, maintaining 74% of its capacity when the *I*_d_ was boosted from 1 to 10 mA cm^−2^. Electrochemical impedance spectroscopy (EIS) revealed that the solution resistance (*R*_s_) and *R*_ct_ of the CC/CuS@PEDOT-10 electrode were 0.82 Ω and 0.39 Ω, respectively, lower than the CC/CuS electrode (*R*_s_ = 1.02 Ω, *R*_ct_ = 0.43 Ω). This indicates that the PEDOT sheath enhances conductivity and reduces internal resistance, facilitating better interfacial electron transfer. The electrode's durability and stability were confirmed through 10 000 GCD cycles at a moderate *I*_d_ of 5 mA cm^−2^ in a 2 M KOH electrolyte. The hybrid electrode retained 98.1% of its initial capacity after cycling, demonstrating exceptional long-term cycling performance. The stability and structure of the electrode were attributed to the ultrathin, highly conductive PEDOT sheath, which aids in fast ion/electron transport and protects the underlying CC/CuS nanoplate arrays from degradation. Post-cycling analysis using field emission scanning electron microscopy (FESEM), X-ray diffraction (XRD), and X-ray photoelectron spectroscopy (XPS) showed that the CuS nanoplates maintained their structural integrity, despite some detachment of the PEDOT sheath. The crystalline structure and XPS spectra remained consistent with those of the uncycled electrode, indicating the robustness of the CC/CuS@PEDOT-10 hybrid structure. Furthermore, a flexible quasi-solid-state asymmetric device as illustrated in Fig. 14 constructed from this electrode delivered a *C*_s_ of 0.331 mA h cm^−2^ at a *I*_d_ of 1 mA cm^−2^. This device achieved a maximum *E*_d_ of 2.21 mW h cm^−3^ at a *P*_d_ of 4.20 mW cm^−3^ and maintained an *E*_d_ of 1.89 mW h cm^−3^ even at a high *P*_d_ of 41.66 mW cm^−3^.

Liu *et al.*^46^ synthesized PPy@Co_0.85_Se nanocomposites using an electrodeposition method. In this composite, Co_0.85_Se nanoparticles were decorated onto conductive PPy nanowires. The PPy@Co_0.85_Se hybrid electrode demonstrated a high capacitive performance, achieving a *C*_s_ of 827 C g^−1^ at *I*_d_ of 1 A g^−1^, which significantly outperformed the pure Co_0.85_Se electrode's *C*_s_ of 282 C g^−1^ at the same *I*_d_. The PPy@Co_0.85_Se electrode retained 67% of its capacity, while the pure Co_0.85_Se electrode retained only 46%. After undergoing 5000 GCD cycles at 10 A g^−1^, the PPy@Co_0.85_Se hybrid electrode retained 93% of its initial capacity, which is significantly higher than the 71% retention observed in the pure Co_0.85_Se electrode. EIS data revealed that the PPy@Co_0.85_Se hybrid electrode had a *R*_s_ of 0.64 Ω and a *R*_ct_ of 0.14 Ω, whereas the pure Co_0.85_Se electrode exhibited higher *R*_s_ and *R*_ct_ values of 2.44 Ω and 1.54 Ω, respectively. The improved performance of the PPy@Co_0.85_Se electrodes can be attributed to several factors. First, the dispersed Co_0.85_Se nanoparticles on PPy nanowires offer better exposure of electroactive sites to electrolyte ions and shorten the ion diffusion path, enhancing the rate capacities of the electrodes. Second, the interconnected PPy nanowires form a conductive network on the Ni foam substrate, providing efficient pathways for fast charge transport and accelerating electrode reactions. Lastly, the PPy nanowires act as a structural backbone, anchoring the Co_0.85_Se nanoparticles and maintaining structural integrity during repeated CD cycles, leading to high initial capacity retention. Additionally, an asymmetric device composed of PPy@Co_0.85_Se and nitrogen-doped carbon nanotube (N-CNT) electrodes was fabricated. This device demonstrated specific capacities of 234, 226, 211, 194, and 171 C g^−1^ at *I*_d_ of 1, 2, 3, 5, and 10 A g^−1^, respectively. The device achieved a maximum *E*_d_ of 51.9 W h kg^−1^ at a *P*_d_ of 812 W kg^−1^ and retained an *E*_d_ of 38.1 W h kg^−1^ at a high *P*_d_ of 7953 W kg^−1^.

**Fig. 15:**
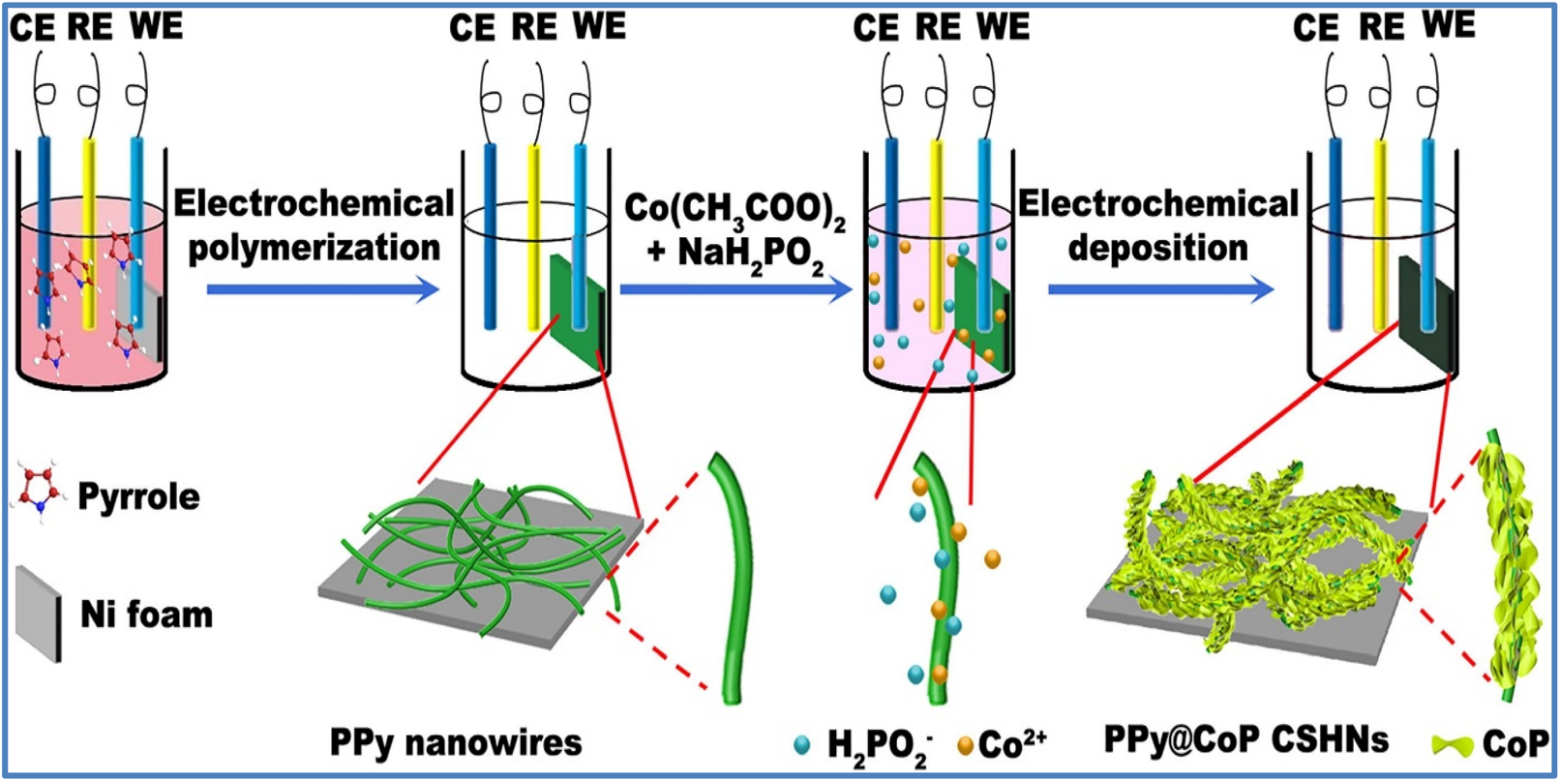
Schematic illustration of preparing the PPy@CoP composite electrode (reproduced from ref. 47 with permission from Elsevier© 2021).

**Fig. 16:**
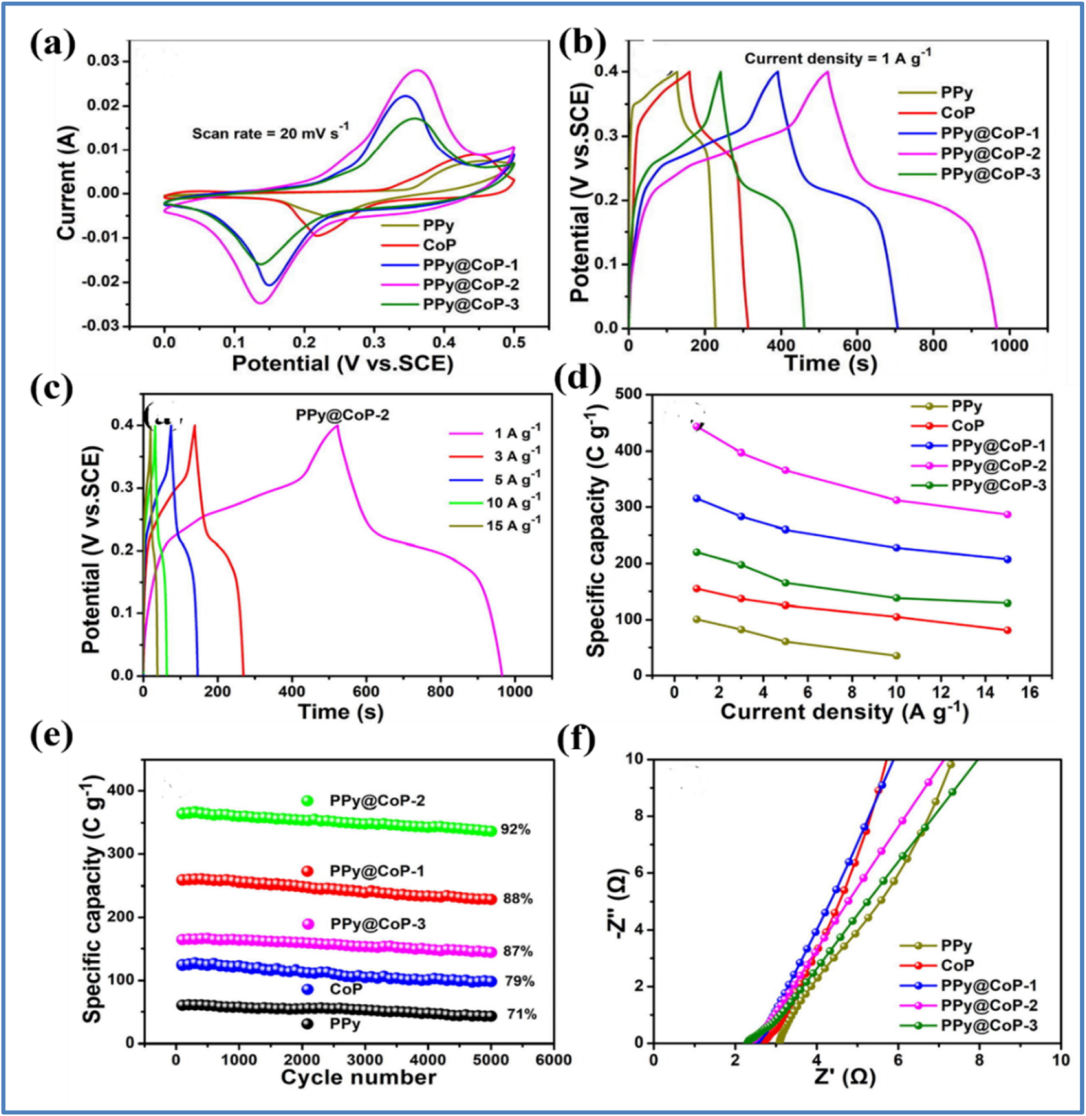
(a) CV curves at 20 mV s^−1^ (b) GCD curves at 1 A g^−1^ for PPy, CoP, PPy@CoP-1, PPy@CoP-2, and PPy@CoP-3 (c) GCD curve of PPy@CoP-2 at various *I*_d_ (d) *C*_s_*vs. I*_d_ (e) stability performance for 5000 cycles, and (f) Nyquist plot for PPy, CoP, PPy@CoP-1, PPy@CoP-2, and PPy@CoP-3 (reproduced from ref. 47 with permission from Elsevier© 2021).

Yue *et al.*^47^ developed a nanostructured composite of PPy@CoP-*x* (*x* = 1, 2, 3) using the electrochemical deposition technique as shown in Fig. 15. The electrochemical studies of PPy, CoP, PPy@CoP-1, PPy@CoP-2, and PPy@CoP-3 electrodes are depicted in Fig. 16. The composite electrode, PPy@CoP-2, achieved a *C*_s_ of 440 C g^−1^ at 1 A g^−1^, which is significantly higher than the specific capacities of the individual components, CoP and PPy, which were 155 C g^−1^ and 101 C g^−1^, respectively. The PPy@CoP-2 electrodes maintained 64.6% of their stability when tested at 15 A g^−1^, outperforming the CoP and PPy electrodes, which retained only 52.5% and 34.9%, respectively.

Furthermore, the PPy@CoP-2 electrodes exhibited a capacity retention of 92% after 5000 GCD cycles at 5 A g^−1^, while the CoP and PPy electrodes retained 79% and 71% of their initial capacities, respectively. In terms of resistance values, the PPy@CoP-2 electrode demonstrated a series resistance (*R*_s_) of 2.34 Ω and a *R*_ct_ of 0.12 Ω. These values are lower compared to the CoP electrode (*R*_s_ of 2.70 Ω and *R*_ct_ of 0.27 Ω) and the PPy electrode (*R*_s_ of 3.09 Ω and *R*_ct_ of 0.33 Ω). For practical applications, a device composed of PPy@CoP-2 and nitrogen-doped carbon nanotubes (N-CNTs) achieved a *C*_s_ of 183 C g^−1^ at 1 A g^−1^. This device demonstrated an *E*_d_ of 38.1 W h kg^−1^ at a *P*_d_ of 750 W kg^−1^, and it maintained an *E*_d_ of 27.8 W h kg^−1^ even at a high *P*_d_ of 7502 W kg^−1^. After 5000 cycles at 5 A g^−1^, the device retained 91% of its capacity and exhibited a *η* of 78%.

**Fig. 17:**
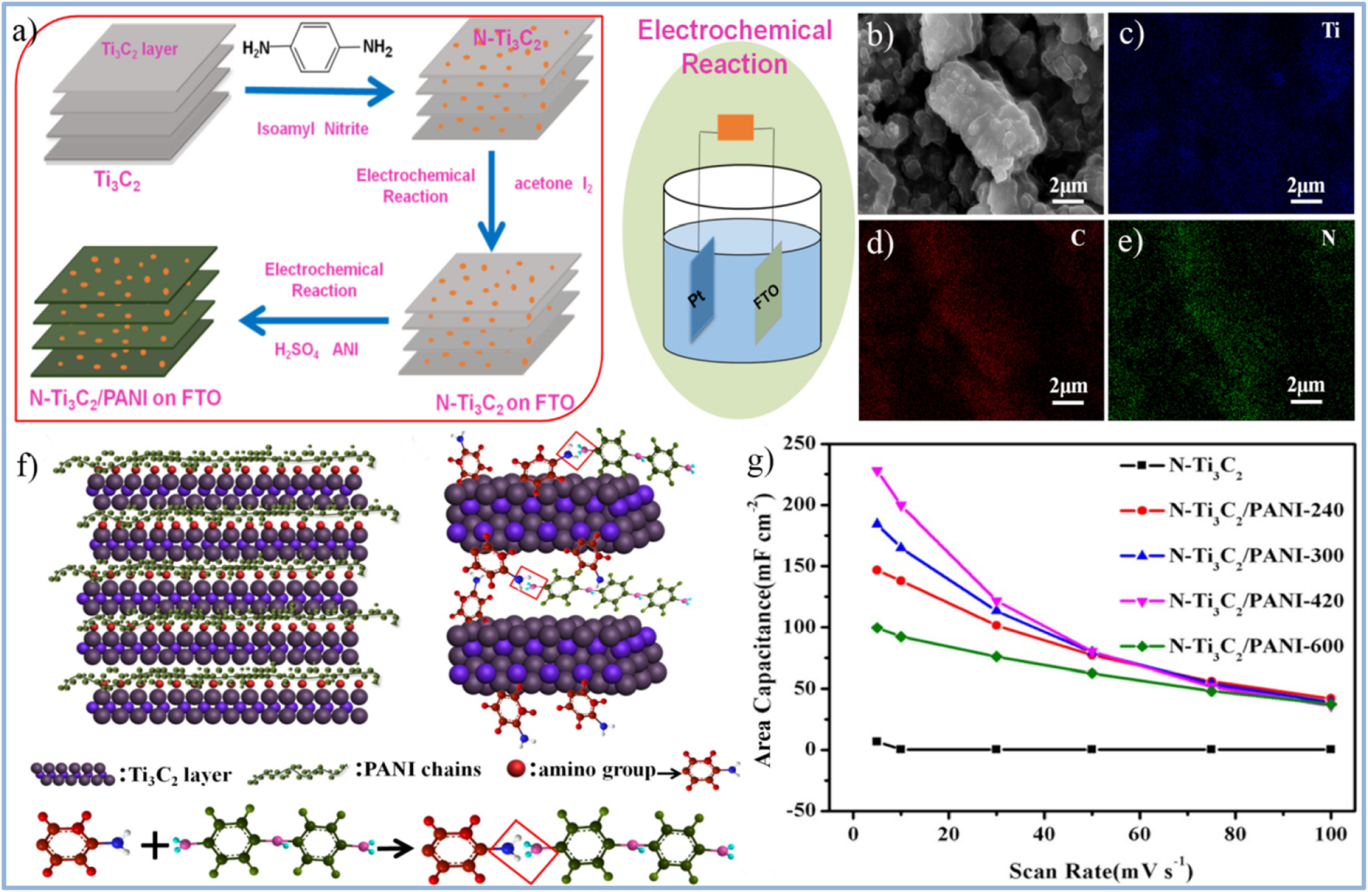
(a) Schematic diagram for the preparation of N-Ti_3_C_2_/PANI. (b) SEM image of N-Ti_3_C_2_/PANI and EDS spectrum (c) Ti, (d) C, and (e) N. (f) Atomic schematic diagram of PANI intercalation N-Ti_3_C_2_. (g) The specific capacitance of pure N-Ti_3_C_2_ and N-Ti_3_C_2_/PANI electrodes from 5–100 mV s^−1^ (reproduced from ref. 48 with permission from Elsevier© 2019).

MXene/conducting polymer composites have shown great promise as advanced electrode materials. Wu *et al.*^48^ successfully synthesized organ-like amino-Ti_3_C_2_ (N-Ti_3_C_2_)/PANI composites using a two-step electrochemical approach. As illustrated in Fig. 17(a), N-Ti_3_C_2_ is first deposited or coated onto an FTO-glass substrate through an electrochemical reaction. Then, the ordered Ti_3_C_2_ MXene structure serves as a scaffold, where the PANI chains are electrochemically polymerized onto the FTO–glass substrate under constant voltage. The SEM image in Fig. 17(b) and the EDS spectra in Fig. 17(c)–(e) confirm the effective integration of N-Ti_3_C_2_ and PANI. Fig. 17(f) highlights the unique bonding mechanism between N-Ti_3_C_2_ and PANI, distinct from the typical direct interaction between Ti_3_C_2_ and PANI. The amine nitrogen on the PANI chain and the amino group on N-Ti_3_C_2_ are tightly bonded through chemical interactions, which enhance the spacing and surface area of the Ti_3_C_2_ MXenes, effectively preventing restacking of MXene sheets. Moreover, the organ-like N-Ti_3_C_2_/PANI composites, formed through covalent grafting, provide a fast and precise channel for ion and charge transfer, boosting the charge transfer rate of the composites. The special structure and bonding mechanism contribute to the excellent electrochemical properties of the N-Ti_3_C_2_/PANI composites. Specifically, in a 0.5 M H_2_SO_4_ electrolyte solution at 5 mV s^−1^, the N-Ti_3_C_2_/PANI composite exhibits outstanding performance, with a maximum surface capacitance of 228 mF g^−1^, which is 32 times greater than that of the pure Ti_3_C_2_ film, as shown in Fig. 17(g). Additionally, the N-Ti_3_C_2_/PANI composite electrode retains 85% of its capacitance after 1000 cycles.

**Fig. 18:**
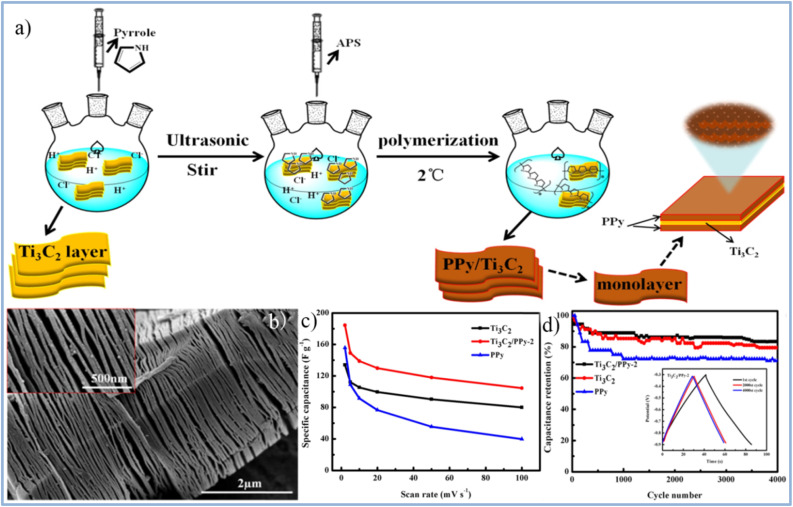
(a) Schematic illustration of preparing Ti_3_C_2_T_*x*_/PPy composites through low-temperature *in situ* polymerization of PPy on Ti_3_C_2_ nanosheets. (b) SEM image of Ti_3_C_2_T_*x*_/PPy (inset of a partially enlarged view). (c) The specific capacitance of PPy, Ti_3_C_2_, and Ti_3_C_2_/PPy at different scanning rates. (d) Cycle curves of PPy, Ti_3_C_2_, and Ti_3_C_2_/PPy at 1 A g^−1^ (reproduced from ref. 49 with permission from Elsevier© 2019).

Wang *et al.*^49^ prepared Ti_3_C_2_T_*x*_/PPy composite material using this method primarily relies on the combination of hydrogen bonds and electrostatic forces between Ti_3_C_2_T_*x*_ nanosheets and PPy chains. Additionally, the intercalation of homogeneous PPy nanoparticles expands the interlayer spacing of Ti_3_C_2_T_*x*_ nanosheets. At the same time, the highly oriented polymer chains provide more channels for charge transfer and electrolyte ion diffusion, thereby enhancing the specific capacitance and reducing charge transfer resistance. Notably, as shown in Fig. 18(c),^49^ the Ti_3_C_2_T_*x*_/PPy composite electrode with the optimal ratio exhibits a specific capacitance of 184.36 F g^−1^ at 2 mV s^−1^, which is 37% higher than the specific capacitance of the pure Ti_3_C_2_T_*x*_ MXene electrode (133.91 F g^−1^). At a current density of 1 A g^−1^, the capacitance of the Ti_3_C_2_T_*x*_/PPy composite electrode retains 83.33% after 4000 charge–discharge cycles, as seen in Fig. 18(d). The enhanced electrochemical performance and cycle stability of the material are attributed to the synergistic effects between Ti_3_C_2_T_*x*_ nanosheets and PPy nanoparticles, along with their complementary energy storage mechanisms. Most importantly, this method provides a low-cost and convenient approach for preparing Ti_3_C_2_T_*x*_/PPy composites on a large scale.^49^

Inal *et al.*^50^ synthesized PEDOT:PSS:MXene films using electrochemical polymerization and co-doping techniques. Compared to thin films composed of a single dopant and PEDOT, the incorporation of PSS and MXene as co-dopants with PEDOT enables a better synergy between the properties of MXene and PEDOT, resulting in a polymer composite with higher specific capacitance and energy density. The PEDOT:PSS:MXene film (607 ± 85.3 F cm^−3^, with a capacity retention rate of 78% after 500 cycles) exhibits a higher capacitance than PEDOT:PSS (195.6 ± 1 F cm^−3^, 37%) and PEDOT:MXene (358.9 ± 16.7 F cm^−3^, 58%), demonstrating superior volume capacitance and cycle stability (Table 2).

**Table 2:** Comparative table of conducting polymer-based binary TMC and their performance

S. no.	Composite material	Formula	Conductivity	Current density	Cyclic stability	References
1	Cobalt-oxide core/shell nanosheet	Co_3_O_4_@PPy	2.11 F cm^−2^	2 mA cm^−2^	85.5% after 5000 cycles	43
2	Nanowire arrays coated with polypyrrole	NiCo_2_O_4_@PPy NWAs	2244.5 F g^−1^	1 A g^−1^	89.5% after 10 000 cycles	44
3	Conducting polyethylenedioxythiophene	CC/CuS@PEDOT-10	1358 F g^−1^	10 mA cm^−2^	96.7% after 10 000 cycles	45
4	PPy@CoP composite electrode	PPy@Co_0.85_Se	827 C g^−1^	1 A g^−1^	93% after 5000 cycles	46
5	Nanostructured composite of PPy@CoP-*x* (*x* = 1, 2, 3)	PPy@CoP-2	183 C g^−1^	5 A g^−1^	92% after 5000 cycles	47
6	Organ-like amino Mxene composites	Ti_3_C_2_ (N-Ti_3_C_2_)/PANI	228 mF g^−1^	1 A g^−1^	85% after 1000 cycles	48
7	MXene were coated with polypyrrole particles	Ti_3_C_2_T_*x*_/PPy	184.36 F g^−1^	1 A g^−1^	83.33% after 4000	49
8	PPy/Ti_3_C_2_T_*x*_ film	PPy/Ti_3_C_2_T_*x*_	1000 F cm^−3^	5 mV s^−1^	92%, 25 000	51

## 3. Ternary transition metal composites for SCs

**Fig. 19:**
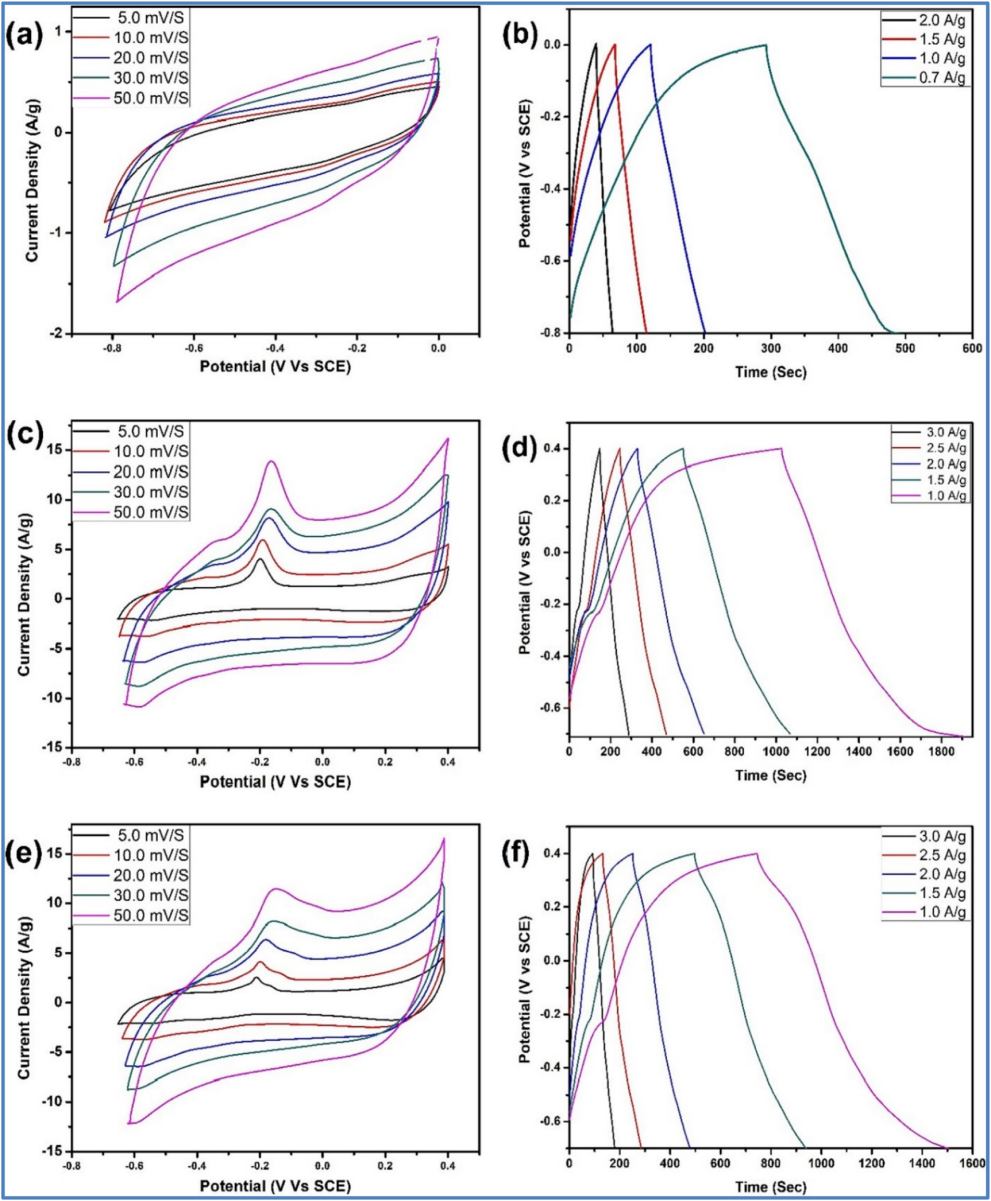
(a), (c), and (e) CV curves at varying *ν* (b), (d), and (f) GCD curves at different *I*_d_ of AC, 25NiO@Co_3_O_4_-AC and 25Co_3_O_4_@NiO-AC electrodes, respectively (reproduced from ref. 52 with permission from Elsevier© 2023).

Ternary composites, composed of three dissimilar materials, often exhibit synergistic properties that exceed those of binary composites. Ternary composites in this category may have different configurations, including transition metals, carbon and conducting polymers. Mostafa S. Gouda *et al.*^52^ synthesized nanocomposites of cobalt oxide-AC and nickel oxide-AC. To enhance electrochemical performance, they prepared these nanocomposites with varying weight percentages (10, 25, 50, and 75 wt%) of nickel oxide and cobalt oxide nanoparticles. The CV curves and GCD curves of AC, 25NiO@Co_3_O_4_-AC, and 25Co_3_O_4_@NiO-AC are given in Fig. 19. The CV curve of AC demonstrated the EDLC behavior whereas the CV curve of composite 25NiO@Co_3_O_4_-AC and 25Co_3_O_4_@NiO-AC clarifying the synergy between EDLC and redox-active nature of 25NiO@Co_3_O_4_ and 25Co_3_O_4_@NiO. The AC electrode showed the lowest *C*_s_ of 105 F g^−1^. In contrast, the nanocomposite electrodes 25NiO@Co_3_O_4_-AC and 25Co_3_O_4_@NiO-AC achieved the highest *C*_s_, 800.9 and 691.8 F g^−1^, respectively, at *I*_d_ of 1 A g^−1^ when tested in 3 M KOH. Their corresponding energy densities were 136.6 and 116.2 W h kg^−1^. A *C*_s_ retention of 98.1% after 5000 GCD cycles at 10 A g^−1^ was reported for the 25Co_3_O_4_@NiO-AC electrode.

Su *et al.*^53^ successfully synthesized an orderly arranged bead-chain ternary nanocomposite consisting of Cu_2_O, Mn_3_O_4_, and NiO (Cu_2_O–Mn_3_O_4_–NiO) through the electrospinning technique. The electrochemical performance of the resulting material was evaluated in a 6 M KOH electrolyte, yielding a maximum *C*_s_ of 1306 F g^−1^ at a *ν* of 5 mV s^−1^. Shahat *et al.*^54^ synthesized a Mn_3_O_4_/TiO_2_/rGO ternary nanocomposite for use in SCs through the hydrothermal method. The electrochemical performance of the material was evaluated in a 6 M KOH electrolyte, where it demonstrated a *C*_s_ of 356 F g^−1^ and retained 91% of its *C*_s_ after 3000 cycles. Additionally, they constructed an ASC, utilizing Mn_3_O_4_/TiO_2_/rGO as the PE and graphene as the NE. This device delivered an *E*_d_ of 31.95 W h kg^−1^ at a *P*_d_ of 718 W kg^−1^ and maintained 87% of its *C*_s_ after 1000 cycles, showcasing excellent cycling stability.

Recently, mixed transition metal sulfides have garnered significant attention from researchers. Among them, NiCo_2_S_4_ has emerged as one of the most widely studied electrode materials, demonstrating exceptional performance. Guo *et al.*^55^ synthesized a ternary composite of CoNi-Layered Double Hydroxide (LDH)/NiCo_2_S_4_/Reduced Graphene Oxide (RGO) using a straightforward one-step hydrothermal deposition method. The study found that NiCo_2_S_4_ nanoparticles were well-dispersed across the RGO surface, while CoNi-LDH was uniformly coated. This distinctive structure facilitates the efficient transfer of charge carriers. The CoNi-LDH/NiCo_2_S_4_/RGO composite showed a *C*_s_ of 1846.66 F g^−1^ at 1 A g^−1^, with 93.57% retention after 5000 cycles. Additionally, the ASC fabricated from this material achieved an *E*_d_ of 28.88 W h kg^−1^. Jia *et al.*^56^ developed NiCo-layered double hydroxide (LDH)/NiCo_2_S_4_ nanotube arrays adorned with black phosphorus quantum dots (BPQD) using a combination of solvothermal synthesis and electrostatic adsorption. The material demonstrated an impressive *C*_s_ of 2938.2 F g^−1^ at a *I*_d_ of 1 A g^−1^. The ASC constructed from this material delivered an *E*_d_ of 133.7 W h kg^−1^ at a *P*_d_ of 800 W kg^−1^ while retaining 76.5% of its *C*_s_ after 10 000 CD cycles.

**Fig. 20:**
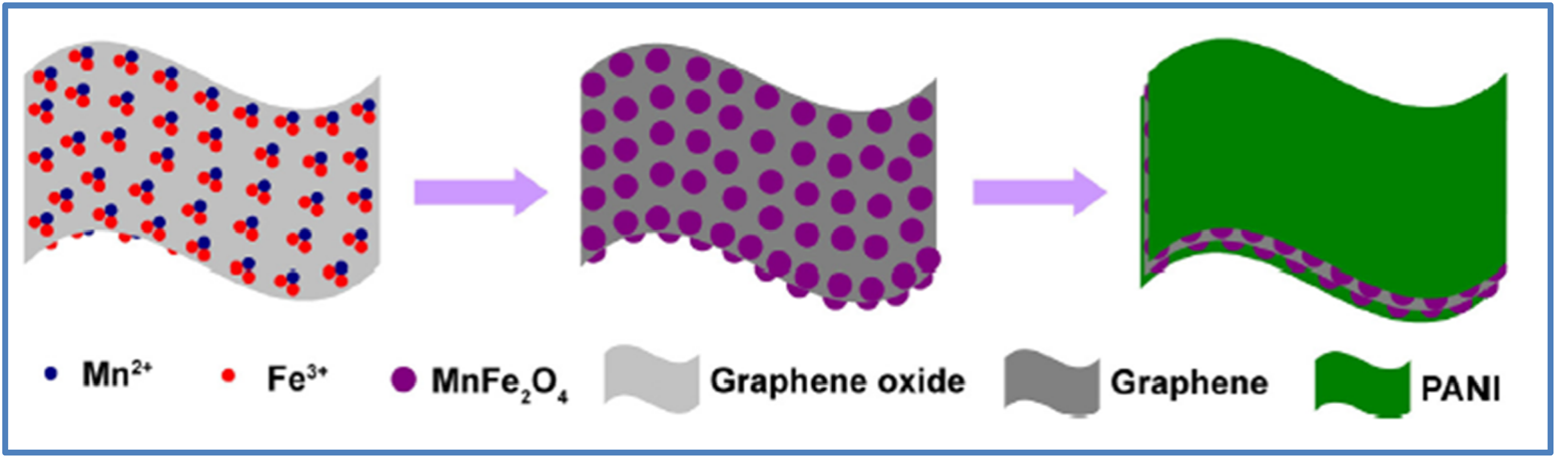
Schematic illustration for the formation of ternary manganese ferrite/graphene/polyaniline nanostructure (reproduced from ref. 57 with permission from Elsevier© 2014).

**Fig. 21:**
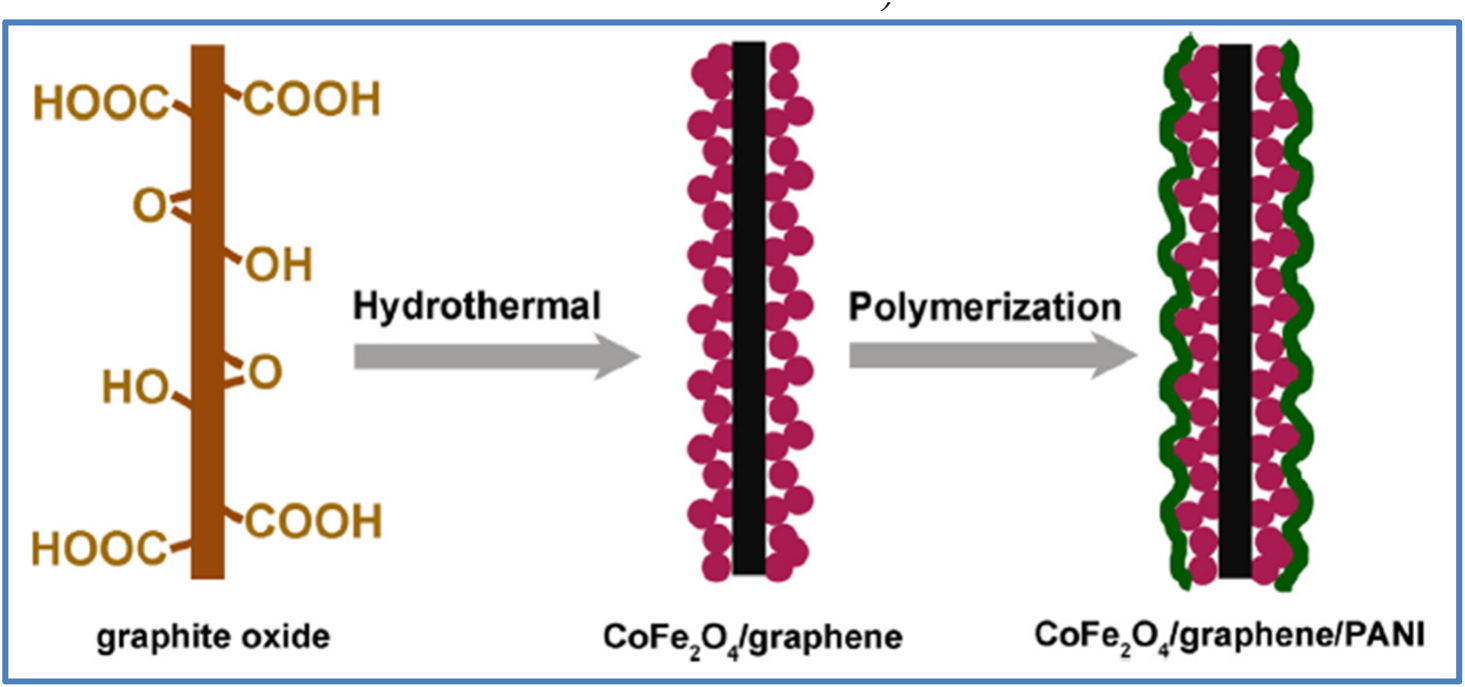
Schematic illustration for the preparation of ternary cobalt ferrite/graphene/polyaniline nanocomposites (reproduced from ref. 59 with permission from Elsevier© 2014).

PANI is one of the most commonly used conducting polymers in SC applications. In this section, we will examine ternary composites incorporating PANI. Xiong *et al.*^57^ explored a manganese ferrite/graphene/polyaniline composite for SC use. The material was synthesized through a two-step process involving hydrothermal treatment followed by polymerization, as illustrated in Fig. 20. Their results showed a *C*_s_ of 454.8 F g^−1^ at a *I*_d_ of 0.2 A g^−1^. Additionally, the material demonstrated strong rate capability, retaining 75.8% of its capacity at 5 A g^−1^, along with 76.4% *C*_s_ retention after 5000 cycles at 2 A g^−1^. Huang *et al.*^58^ developed a MnO_2_/PANI/MWCNTs ternary nanocomposite for SCs. This composite was evaluated in a 1 M KOH electrolyte, where it achieved a maximum *C*_s_ of 395 F g^−1^. Furthermore, the material retained 72% of its *C*_s_ after 1000 CD cycles at *I*_d_ of 1 A g^−1^. Xiong *et al.*^59^ developed a cobalt ferrite/graphene/polyaniline ternary nanocomposite through a two-step process involving hydrothermal synthesis followed by *in situ* polymerization, as illustrated in Fig. 21. The electrochemical analysis revealed a *C*_s_ of 1133.3 F g^−1^ at a *ν* of 1 mV s^−1^, and 767.7 F g^−1^ at a *I*_d_ of 0.1 A g^−1^ in a three-electrode system. The material also demonstrated excellent cycling stability, retaining 96% of its *C*_s_ after 5000 cycles. Wang *et al.*^60^ synthesized a nitrogen-doped graphene/nickel ferrite/polyaniline (NGNP) ternary nanocomposite through a two-step process involving hydrothermal synthesis and polymerization. The composite exhibited a *C*_s_ of 645.0 F g^−1^ at a *ν* of 1 mV s^−1^. When tested in a two-electrode symmetric configuration, the device delivered an *E*_d_ of 23.2 W h kg^−1^ at a *P*_d_ of 27.7 W kg^−1^. The material demonstrated excellent cycling stability, with only a 5% drop in *C*_s_ after 5000 cycles and a 10% drop after 10 000 cycles, indicating its potential for SC applications. Shafi *et al.*^61^ developed a ternary composite consisting of LaMnO_3_, reduced graphene oxide (RGO), and polyaniline (PANI) using an *in situ* polymerization method. This composite was utilized to construct two types of SCs: a SSC (LaMnO_3_/RGO/PANI//LaMnO_3_/RGO/PANI) and an ASC (LaMnO_3_/RGO/PANI//RGO). The ASC demonstrated a *C*_s_ of 111 F g^−1^ at *I*_d_ of 2.5 A g^−1^, maintaining over 50% *C*_s_ retention at a higher *I*_d_ of 20 A g^−1^. Additionally, this device achieved a maximum *E*_d_ of 50 W h kg^−1^ at a *P*_d_ of 2.25 kW kg^−1^. Maity *et al.*^62^ developed a composite material consisting of boron nitride (BN), CNTs, and PANI using a simple synthesis method. This composite exhibited a *C*_s_ of 387.5 F g^−1^ at *I*_d_ of 1 A g^−1^ when tested in a 1 M KCl electrolyte solution. The BN/CNT/PANI composite demonstrated an impressive *E*_d_ of 34.44 W h kg^−1^ at a *P*_d_ of 400 W kg^−1^. Furthermore, the composite maintained an 87% *C*_s_ retention after 600 CD cycles, showcasing its excellent cycling stability.

**Fig. 22:**
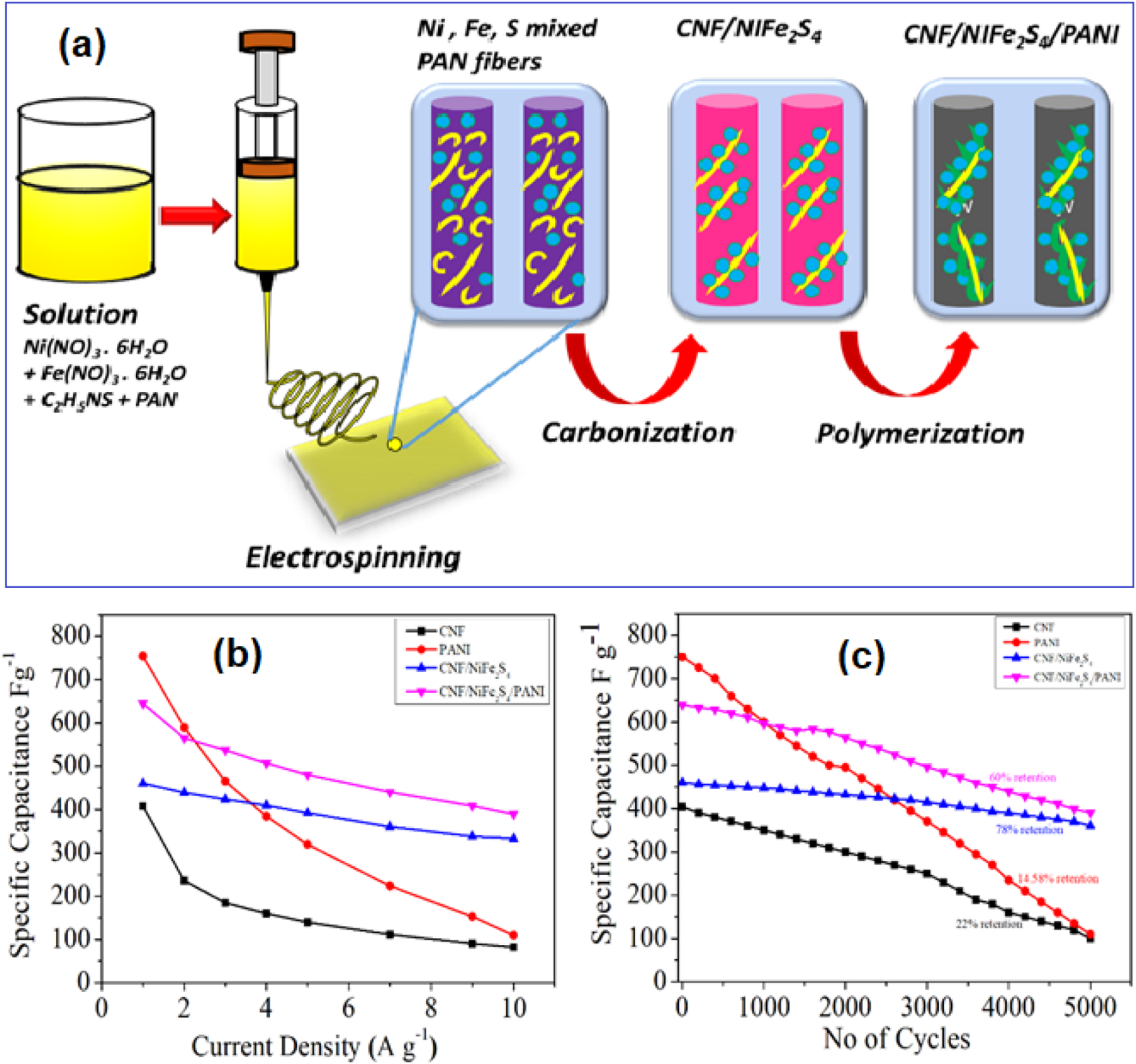
(a) Schematic representation for the preparation of CNF/NiFe_2_S_4_/PANI (b) *I*_d_*vs. C*_s_ curve (c) cycling stability of CNF, PANI, CNF/NiFe_2_S_4_, and CNF/NiFe_2_S_4_/PANI (reproduced from ref. 63 with permission from Elsevier© 2021).

Atram *et al.*^63^ synthesized a novel ternary nanocomposite, CNF/NiFe_2_S_4_/PANI, using a combination of electrospinning and *in situ* polymerization techniques. The preparation method and electrochemical results for this composite are illustrated in Fig. 22. The CV curve for CNF/NiFe_2_S_4_/PANI showed a significantly larger enclosed area compared to CNF, PANI, and CNF/NiFe_2_S_4_ at the same *ν* of 10 mV s^−1^, indicating a superior capacitive performance. This enhancement is attributed to the increased number of active sites in the CNF/NiFe_2_S_4_/PANI structure. The ternary nanocomposite achieved a maximum *C*_s_ of 645 F g^−1^ at a *I*_d_ of 1 A g^−1^, outperforming the CNF/NiFe_2_S_4_ binary composite, which exhibited a *C*_s_ of 460 F g^−1^ at the same *I*_d_. Additionally, it demonstrated excellent cycling stability, retaining 60% of its *C*_s_ after 5000 cycles. The composite also delivered a maximum *E*_d_ of 22.38 W h kg^−1^ at a *P*_d_ of 125 W kg^−1^. Raza *et al.*^64^ fabricated a composite of NiCo_2_S_4_, g-C_3_N_4_, and PANI (NiCo_2_S_4_/g-C_3_N_4_/PANI) using a combination of hydrothermal synthesis, thermal condensation, and chemical oxidative polymerization techniques. The resulting electrode exhibited a *C*_a_ of 3.4 F cm^−2^ (equivalent to 1799.07 F g^−1^) and demonstrated impressive stability, retaining 90% of its *C*_a_ after 2700 cycles at a *I*_d_ of 2 mA cm^−2^. The material's rate capability showed a gradual decline, with *C*_a_ retention of 87.36% at 5 mA cm^−2^, 82.92% at 10 mA cm^−2^, and 50.35% at a higher *I*_d_ of 20 mA cm^−2^.

Karim *et al.*^65^ developed a novel ternary electrode material for SCs by sonochemically synthesizing CNTs-PANI/CoNi(PO_4_)_2_ nanocomposites. The incorporation of the phosphate group in this ternary system is a unique approach, as the use of phosphates in SC electrode materials is relatively rare. The resulting nanocomposites exhibited an impressive *C*_s_ of 1268 C g^−1^ (2136 F g^−1^) and demonstrated excellent diffusive behavior. Furthermore, they fabricated a HSC device using the CNTs-PANI/CoNi(PO_4_)_2_ nanocomposites as the PE and AC as the NE. This device showcased an outstanding *E*_d_ of 87 W h kg^−1^ and a high *P*_d_ of 680 W kg^−1^. Remarkably, the device maintained an excellent cyclic performance, retaining nearly 100% of its capacity after 5000 CD cycles. The synergistic effects of the various components in the ternary nanocomposite, namely the CNT, PANI, and CoNi(PO_4_)_2_, contributed to the enhanced electrochemical performance of the SC.

PPy is another promising material for SC electrodes due to its high energy storage capacity, excellent electrical conductivity through doping and de-doping processes, ease of synthesis, and stability. Wang *et al.*^66^ synthesized a graphene/SnO_2_/PPy (GSP) nanocomposite electrode *via* a one-pot *in situ* polymerization method. When tested in a 1 M H_2_SO_4_ electrolyte, the material demonstrated a high *C*_s_ of 616 F g^−1^ at a *ν* of 1 mV s^−1^, along with an *E*_d_ of 19.4 W h kg^−1^ and a *P*_d_ of 9973.26 W kg^−1^. In another study, Oraon *et al.*^67^ developed a nanoclay-based ternary graphene/PPy nanocomposite using both *in situ* and *ex situ* polymerization techniques. When tested in 1 M KCl with a potential window of 0 to 0.8 V, the composite produced *via in situ* polymerization showed a *C*_s_ of 347 F g^−1^ at a *ν* of 10 mV s^−1^, outperforming the *ex situ* composite. This research highlights the role of nanoclay in enhancing *C*_s_, demonstrating the potential of nanocomposites for SCs. Similarly, Ishaq *et al.*^68^ investigated a ternary composite made of graphene, doped metal oxides (iron oxide), and PPy, revealing *C*_s_ of 147 F g^−1^ for an rGO/MnFe_2_O_4_ binary composite and 232 F g^−1^ for the rGO/MnFe_2_O_4_/PPy ternary composite. The improvement in *C*_s_ for the ternary material showcases the synergistic effect of the PPy additives (Table 3).

**Table 3:** Comparative table of ternary transition metal composites for SCs

S. no.	Composite material	Formula	Conductivity	Current density	Cyclic stability	References
1	Nanocomposites of cobalt oxide-AC and nickel oxide-AC	25NiO@Co_3_O_4_-AC	691.8 F g^−1^	1 A g^−1^	98.1% after 5000 cycles	52
2	Bead-chain ternary nanocomposite	Cu_2_O–Mn_3_O_4_–NiO	1306 F g^−1^	5 mV s^−1^	96% after 3000 cycles	53
3	Mn–Ti with rGO ternary nanocomposite	Mn_3_O_4_/TiO_2_/rGO	356 F g^−1^	1 A g^−1^	91% after 10 000 cycles	54
4	CoNi-layered double hydroxide	CoNi-LDH/NiCo_2_S_4_/RGO	1846.66 F g^−1^	1 A g^−1^	93.57% after 5000 cycles	55
5	NiCo-layered double hydroxide nanotube arrays	NiCo_2_S_4_	2938.2 F g^−1^	1 A g^−1^	76.5% after 10 000 cycles	56
6	Manganese ferrite/graphene/polyaniline composite	MnFe_2_O_4_/PANI/MWCNTs	454.8 F g^−1^	5 A g^−1^	76.4% after 5000 cycles	57
7	Manganese oxide/polyaniline/MW carbon nanotube based ternary nanocomposite	MnO_2_/PANI/MWCNTs	395 F g^−1^	1 A g^−1^	72% after 1000 cycles	58
8	Cobalt ferrite/graphene/polyaniline ternary nanocomposite	CoFe_2_O_4_/G/PANI	1133.3 F g^−1^	1 mV s^−1^	96% after 5000 cycles	59
			767.7 F g^−1^	0.1 A g^−1^		
9	Boron nitride (BN), CNTs, and PANI based composite	BN/CNT/PANI	387.5 F g^−1^	1 A g^−1^	87% after 600 cycles	62
10	Novel ternary nanocomposite	CNF/NiFe_2_S_4_/PANI	645 F g^−1^	1 A g^−1^	60% after 5000 cycles	63
11	Composite of NiCo_2_S_4_, g-C_3_N_4_, and PANI	NiCo_2_S_4_/g-C_3_N_4_/PANI	1799.07 F g^−1^	1 A g^−1^	90% after 2700 cycles	64
12	Novel ternary electrode material	CNTs-PANI/CoNi(PO_4_)_2_	2136 F g^−1^	1 A g^−1^	100% after 5000 cycles	65
13	Graphene/SnO_2_/PPy (GSP) nanocomposite	SnO_2_/G/PPy	616 F g^−1^	1 mV s^−1^	90% after 5000 cycles	66
14	Zinc-ion hybrid capacitors (ZHCs)	Zn^2+^/CF_3_SO_3_/NHPCs-700	253 mA h g^−1^	0.2 A g^−1^	94% after 200 000 cycles	69
15	Co-doped W_18_O_49_ on carbon cloth (CC)	Co-doped W_18_O_49_/CC	792 F g^−1^	1.0 A g^−1^	90% after 10 000 cycles	70
16	Nickle cobalt oxide with ZnO–CuO composite based nano-structures and nickel foam	NiCo_2_O_4_/ZnO–CuO/NF	3614.8 F g^−1^	2 A g^−1^	98% after 40 000 cycles	71

## 4. Summary and future scope

In this paper, we explored the potential composite materials as energy storage electrode that can be fabricated by combining transition metal oxides/hydroxides, chalcogenides, and phosphides/phosphates with carbon and/or conducting polymers to form binary and ternary composites for SC applications. This paper demonstrated that these composite materials as energy storage electrode exhibit superior electrochemical performance compared to their respective individual. This enhancement is attributed to the synergistic effects that not only create efficient pathways for electron and ion transfer but also ensure the structural integrity and stability of the entire electrode. Although binary composite materials have been well-explored, there is still significant untapped potential in a new class of metal compounds, specifically metal phosphides and phosphates. These materials, when combined with carbon, conducting polymers, or other phosphates/phosphides, remain largely unexplored. Due to the lower electronegativity of phosphorus, these compounds exhibit inherent properties that could contribute to high *C*_s_ and *E*_d_ in SCs. Researchers are encouraged to investigate the potential of such composites. In the realm of ternary composites, research has predominantly focused on metal oxides, leaving other groups such as metal chalcogenides, metal phosphides, and phosphates relatively unexplored. Considering the synergistic advantages that can arise from combining different electrode materials, there is a compelling opportunity to study ternary composites involving these less-explored compounds for SC applications. The fabrication of composite materials as energy storage electrode typically involves multiple steps (often 2–3), which can reduce cost-effectiveness and make the process time-consuming. Researchers are encouraged to explore methods like electrospinning and solvothermal synthesis, which can produce binary composites in a single step. These methods can also streamline the preparation of ternary composites, making the process more sustainable, energy efficient and cost-effective.

## 5. Conclusion

The transition metal-based composites represent a highly promising class of materials for the development of high-performance supercapacitors. The ability to combine multiple materials within binary and ternary composites allows for the synergistic enhancement of electrochemical properties, resulting in superior energy storage capabilities compared to conventional single-material electrodes. These composites offer advantages such as improved energy density, extended cycle life, and a broader voltage window, which are critical for meeting the growing demand for efficient and durable energy storage systems. However, challenges remain in optimizing the synthesis, stability, and scalability of these materials for commercial applications. Future research should focus on overcoming these obstacles by exploring novel material combinations, optimizing fabrication methods, and understanding the long-term performance under real-world conditions. With continued advancements, transition metal-based composites have the potential to play a pivotal role in the future of high-performance energy storage systems, contributing to the development of more efficient, reliable, and sustainable technologies.

## Data availability

This review article is a comprehensive synthesis of previously published literature and does not include original experimental data. All data and information discussed in the manuscript are sourced from publicly available publications, which are appropriately cited throughout the text. As such, no primary data generated or analyzed for the purpose of this review article are available.

## Author contributions

Jannatun Zia – writing original draft, conceptualization, review, and methodology, while M. S. S. R. Tejaswini was responsible for writing comparative tables, reviewing, visualization, and editing the article.

## Conflicts of interest

The authors declare no competing interest.
